# A new framework for disentangling different components of excess mortality applied to Dutch care home residents during Covid-19

**DOI:** 10.1186/s12874-025-02579-1

**Published:** 2025-05-10

**Authors:** Marije H. Sluiskes, Eva A. S. Koster, Jelle J. Goeman, Mar Rodríguez-Girondo, Hein Putter, Liesbeth C. de Wreede

**Affiliations:** 1 Medical Statistics, Biomedical Data Sciences, Leiden University Medical Center, Leiden, The Netherlands; 2 Mathematical Institute, Leiden University, Leiden, The Netherlands

**Keywords:** Relative survival, Excess mortality, Additive hazards, Covid-19, Care homes

## Abstract

**Background:**

Vulnerable subgroups of the population, such as care home residents, often face elevated mortality risks during crises like pandemics or wars. To correctly model and interpret the excess mortality of vulnerable groups during crises, a distinction must be made between the pre-existing heightened mortality of the vulnerable group, the general population’s excess mortality during the crisis, and the crisis-specific excess mortality unique to the vulnerable group.

**Methods:**

We introduce the concept of “excess excess” mortality, which captures the extra excess mortality experienced by vulnerable groups during crises, beyond what can be explained by their excess mortality due to being vulnerable and general population excess mortality. Using individual-level data from Statistics Netherlands, we model the excess excess mortality of Dutch care home residents aged 70 and older during the Covid-19 pandemic. We extend standard relative survival methods by incorporating multiple excess mortality components and use an additive hazards model to accommodate periods of negative excess hazard.

**Results:**

The findings confirm the severe impact of the Covid-19 pandemic on care home residents. In general, men and older age groups experienced higher excess excess mortality, both in absolute and relative terms.

**Conclusions:**

Our approach offers a new perspective on how to model and interpret excess mortality in vulnerable groups during a crisis and provides a methodological foundation for investigating excess excess mortality in other contexts.

**Supplementary Information:**

The online version contains supplementary material available at 10.1186/s12874-025-02579-1.

## Background

Vulnerable subgroups of the general population, such as care home residents or individuals with severe mental health disorders, face heightened mortality risks compared to age- and sex-matched members of the general population [1, 2]. During crises such as wars or pandemics, the general population itself may also experience excess mortality, defined as a higher-than-expected mortality relative to pre-crisis levels. Notable examples include the global Covid-19 pandemic [3], the Spanish Flu [4] and World War II [5].

It is often reasonable to expect that vulnerable subgroups have a higher excess mortality than the general population during such crisis periods, as they are more frail. When investigating whether a vulnerable group has experienced more excess mortality than the general population during a crisis, it is critical to make a proper comparison. This requires accounting for both the pre-existing heightened mortality risk of the vulnerable group as well as the excess mortality experienced by the general population as a whole during the crisis. After adjusting for these two excess mortality components, any remaining excess mortality is then indicative of the excess mortality specific to the vulnerable group during the crisis. We define this remaining component as “excess excess” mortality. Disentangling the different excess mortality components of vulnerable groups during crises is crucial to avoid misinterpretation. Without clearly defining each component, one might for instance incorrectly conflate the excess mortality due to being vulnerable with the excess mortality specifically attributable to the crisis, or one might forget to account for the fact that the population as a whole also experienced excess mortality.

In this paper we focus on modeling the excess mortality of Dutch care home residents during the Covid-19 pandemic years 2020 and 2021. Care home residents, a particularly vulnerable subgroup of the general population, were hit hard by the pandemic [6–10]. Not only did their frailty make them more prone to dying due to the virus or indirect pandemic effects, the nature of care homes makes social distancing or self-isolation very difficult: residents live close to one another, are all seen by the same care givers and generally require frequent care. It is therefore likely that they suffered more during the Covid-19 pandemic period than the general population, and hence experienced excess excess mortality.

Obtaining an accurate picture of the excess excess mortality experienced by care home residents during the Covid-19 pandemic is critical for several reasons. Firstly, it quantifies anecdotal evidence, which was ubiquitous during the pandemic, with numbers and statistics to illustrate the pandemic’s true death toll. Furthermore, it helps policy makers and care givers in preparing for a next pandemic: groups that experienced a particularly high excess mortality rate during the Covid-19 crisis should perhaps be subjected to different protective policy measures in a future pandemic. The excess excess mortality concept introduced in this paper can provide further support to earlier findings and illuminate the dynamics of the underlying individual components of the overall excess mortality in care home residents during the pandemic.

This paper discusses the novel concept of excess excess mortality in detail and presents a methodological framework for disentangling the different components of excess mortality of vulnerable groups during a crisis. Concretely, it illustrates how distinct excess hazards can be estimated if there is more than one cause or indicator of excess mortality (in our motivating application: care home residency and the Covid-19 pandemic). To disentangle the excess mortality components, we extend relative survival methods, which are typically used to model excess mortality. Relative survival models partition the observed overall mortality hazard into a background and an excess component. The background component is generally assumed to be known by taking this hazard from external population life tables [11]. Existing relative survival models are limited to modeling a single excess mortality component, which does not suffice for our motivating example. We hence incorporate multiple excess mortality components in our relative survival model, which, to the best of our knowledge, has not previously been done. Although covariates affecting the excess hazard component are usually assumed to have a proportional effect in relative survival, we assume an additive relationship. Unlike the traditional (Cox) proportional hazards approach, an additive hazards framework accommodates negative excess hazards, which occur when mortality falls below expected levels. Negative hazards were observed during certain periods of the Covid-19 pandemic [3]. We also discuss how our model differs from standard relative survival applications in terms of the source of the background mortality and the model timescale. Finally, we apply our model to individual-level data from Statistics Netherlands to estimate the excess excess hazard of care home residents aged 70 and older in the Netherlands during the Covid-19 pandemic.

Although our motivating example focuses on care home residents during the Covid-19 pandemic, the concept of excess excess mortality is general and can be applied to any setting in which there are two distinct causes of excess mortality, with a possible interaction between these causes, resulting in more or less excess mortality than when considering these causes separately.

## Methods

This section first discusses standard relative survival models in some detail. Next, the concept of excess excess mortality, and how it relates to conventional relative survival, is introduced. Finally, the choices that must be made when fitting an (excess) excess mortality model are described, including a justification of the choices that we made for our motivating application.

### Relative survival models

A major aim of relative survival models is to obtain an estimate of the excess mortality of a certain group, of whom all members share a certain characteristic that results in excess mortality (e.g. because they all suffer from some serious disease). This is achieved by splitting the overall mortality hazard of the group of interest in two cause-specific hazards: a background hazard (also known as a population hazard, as it is typically based on the overall mortality in the general population) and an excess hazard.

**Fig. 1:**
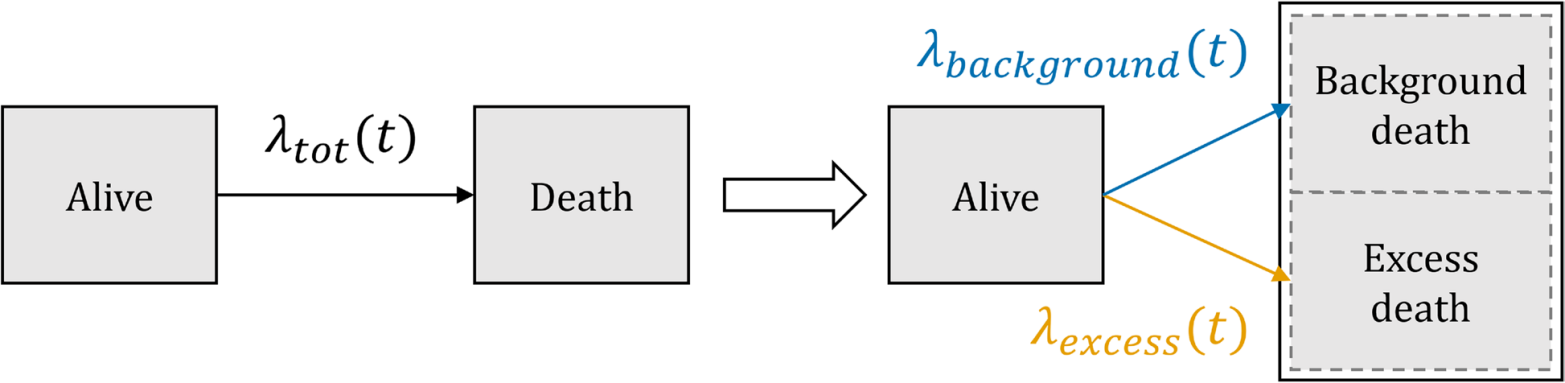
A relative survival model as a competing risk model. Left of the white arrow, it is shown what can be directly derived from the data: overall mortality hazard $\lambda _{tot}(t)$. Relative survival models assume that $\lambda _{tot}(t)$ can be split up as $\lambda _{background}(t) + \lambda _{excess}(t)$ (right of the white arrow)

The structure of relative survival models is generally assumed to be additive, i.e. $\lambda _{tot}(t)$, the total observed hazard at time *t*, is equal to the background hazard plus the excess hazard, $\lambda _{background}(t) + \lambda _{excess}(t)$. Hence, relative survival models are similar in structure to competing risks models, where individuals are at risk of experiencing one of several different events. The difference is that in a competing risk setting, it is known which of the competing events non-censored individuals experienced (e.g. death due to a background mortality cause or an excess mortality cause). In the relative survival setting the cause of death for non-censored individuals is not known: it is only known who died when, but not what of. Figure 1 visualizes the standard relative survival setting. $\lambda _{tot}(t)$, in black, is observed: it entails all deaths. $\lambda _{background}(t)$, in blue, is typically not derived from the data but taken from population life tables, stratified by year, age and sex [11]. The excess hazard $\lambda _{excess}(t)$, in orange, is hence identifiable: $\lambda _{tot}(t)$ is observed and $\lambda _{background}(t)$ is known.

To justify the use of existing life tables for $\lambda _{background}(t)$, the population under study must be similar (conditional on sex and age) to the population used for the background hazard, except for the characteristic or circumstance that results in the excess mortality. Another important assumption that lies behind the use of existing life tables for $\lambda _{background}(t)$ is that the excess hazard of the group under study, $\lambda _{excess}(t)$, contributes little to the overall hazard of the general population. If this is not the case, it is not reasonable to use population life tables for the background hazard $\lambda _{background}(t)$: using the population life tables will result in a higher $\lambda _{background}(t)$ than when only considering those not belonging to the vulnerable group. This inflated background hazard will in turn result in a deflated, and hence biased, estimate of $\lambda _{excess}(t)$.

In principle hazards cannot be negative, as they represent the instantaneous probabilities at which events occur. However, the excess hazard $\lambda _{excess}(t)$ *can* be negative, as this entity is in fact not a true hazard: it is the difference between the quantities $\lambda _{tot}(t)$ and $\lambda _{background}(t)$, which both represent true hazards. Negative excess hazards occur when the observed overall mortality hazard $\lambda _{tot}(t)$ is lower than the background mortality hazard $\lambda _{background}(t)$, i.e. when fewer deaths are observed than expected.

It is often desirable to model the effect of a vector of covariates $\textbf{Z}(t)$ on the excess hazard. These covariates can consist of demographic variables, $\textbf{D}(t)$, as well as variables specific to the group that experiences the excess hazard, $\textbf{W}(t)$: $\textbf{Z}(t) = (\textbf{D}(t),\textbf{W}(t))$. Covariate values may vary over time; however, as in our application the follow-up time is divided in different intervals, where in each interval the variables are assumed to be constant, the time-dependency is dropped from the notation in the remainder of this paper. The background hazard $\lambda _{background}(t)$ only depends on $\textbf{D}$. The excess mortality hazard $\lambda _{excess}(t)$ depends on both $\textbf{D}$ and $\textbf{W}$. When modeling the excess mortality due to some disease, $\textbf{W}$ can for example contain characteristics of this disease or its treatment.

### Excess excess mortality

Standard relative survival models target a single excess mortality component: relative to some background mortality, all extra mortality is considered excess. However, in certain scenarios there can be more than one characteristic or circumstance associated with excess mortality. For instance, in our motivating application, we are interested in modeling the excess mortality of care home residents during Covid-19. Both care home residency and the Covid-19 pandemic are characteristics/circumstances associated with excess mortality. For simplicity, we will henceforth refer to these indicators as ‘causes’ of excess mortality, in line with the cause-specific hazards terminology, but this does not necessarily imply a causal association.

For each individual cause, its associated excess hazard could be determined with a conventional relative survival model. However, it is possible that in care home residents during the Covid-19 pandemic, the sum of these two individual excess hazards does not match the total excess hazard of this group: after all, it is likely that they experienced more excess mortality during the pandemic than the general population, even when taking their already existing excess hazard due to being a care home resident into account. We call this remaining excess hazard the excess excess hazard. In essence, this is the excess hazard that results from the interaction between two (or more) individual causes of excess mortality.

To generalize this: imagine two causes that individually result in excess hazard, *A* and *B*. Those who experience these two causes simultaneously no longer have one excess mortality component, but three: the excess hazard due to cause *A*, $\lambda _{A}(t)$, the excess hazard experienced due to cause *B*, $\lambda _{B}(t)$, and the excess excess hazard component, $\lambda _{A, B}(t)$, capturing any remaining excess hazard:

1 $$\begin{aligned} \lambda _{tot}(t|\textbf{Z}) = \lambda _{background}(t| \textbf{Z}) + \lambda _{A}(t|\textbf{Z}) + \lambda _{B}(t|\textbf{Z}) + \lambda _{A,B}(t|\textbf{Z}). \end{aligned}$$

Again, only $\lambda _{tot}(t|\textbf{Z})$ is known from the data. $\lambda _{tot}(t|\textbf{Z})$ and $\lambda _{background}(t|\textbf{Z})$ are real hazards and must hence take positive values. All three excess hazard components can in principle also be negative.

Causes *A* and *B* in the excess excess mortality model given by Eq. (1) can represent any characteristics likely to result in excess mortality (e.g. different comorbidities). In this paper, we use this model to disentangle the different hazards of a vulnerable group (cause *A*) during a period of crisis (cause *B*). Vulnerable groups during a crisis experience the following hazards: the excess hazard due to belonging to a vulnerable group $\lambda _{vul}(t)$, the excess hazard experienced by the entire population due to a crisis $\lambda _{cri}(t)$, and the excess excess hazard component $\lambda _{vul, cri}(t)$. The excess excess mortality can be interpreted as the excess mortality due to the crisis that is specific to the vulnerable group of interest.

The components $\lambda _{vul}(t)$ and $\lambda _{vul, cri}(t)$ are vulnerable group-specific and hence depend on both $\textbf{D}$ and $\textbf{W}$, whereas $\lambda _{cri}(t)$ and $\lambda _{background}(t)$ hold for the general population and hence only depend on $\textbf{D}$. The total hazard for this group can thus be written as follows:

2 $$\begin{aligned} \lambda _{tot}(t|\textbf{D},\textbf{W}) = \lambda _{background}(t| \textbf{D}) + \lambda _{vul}(t|\textbf{D},\textbf{W}) + \lambda _{cri}(t|\textbf{D}) + \lambda _{vul, cri}(t|\textbf{D},\textbf{W}). \end{aligned}$$

**Fig. 2:**
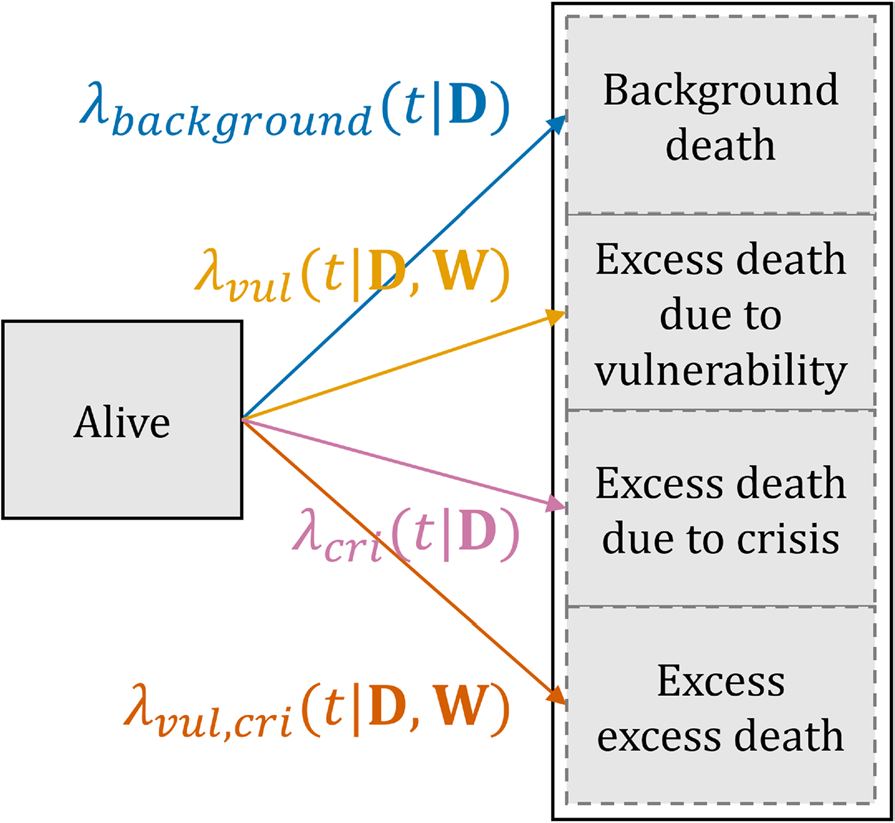
The excess mortality model as a competing risks model. The total observed mortality hazard $\lambda _{tot}$ is the sum of all four components

Figure 2 reflects the situation described by Eq. (2).

It is insightful to consider the different groups that these four hazards describe. Indicator variables can be used to denote whether or not someone belongs to the vulnerable group of interest, and whether or not someone is observed during the crisis of interest. With these two binary contrasts, four different groups can be distinguished: vulnerable yes/no, crisis yes/no. Each group experiences a different combination of hazards; only the members of the vulnerable group in a crisis (group yes/yes) are subjected to the excess excess hazard $\lambda _{vul, cri}(t|\textbf{D},\textbf{W})$. Table 1 provides an overview of the four groups and their corresponding hazard combinations. From this table it also becomes clear how different contrasts can be drawn to derive different hazard components. For instance, the excess hazard experienced by the non-vulnerable population due to the crisis, $\lambda _{cri}(t|\textbf{D})$, can be derived by comparing the overall mortality hazard of the non-vulnerable group before the crisis period (corresponding to $\lambda _{background}(t|\textbf{D})$) with the overall mortality hazard of that same group during the crisis: the difference (consisting of direct crisis effects, indirect crisis effects and factors coinciding with the crisis, such as extreme weather) is captured by $\lambda _{cri}(t|\textbf{D})$.

**Table 1:**
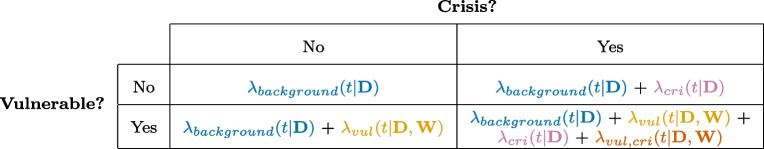
The different components of the excess mortality model in a table. Two different contrasts can be drawn (vulnerable yes/no, crisis yes/no). Each contrast corresponds to a certain group, which experiences certain hazards

From drawing these contrasts, another defining characteristic of our model becomes apparent: whereas conventional relative survival models tend to be used to compare a specific group with the general population *in the same time period*, our model also compares the same group *in different time periods*. This is because vulnerable group membership is a personal characteristic, while the crisis is a temporal one. Hence, contrasts are needed both between different groups in the same time period (to model the difference in (excess) mortality between those belonging to the vulnerable group and those belonging to the rest of the population), as well as within the same groups in different time periods (to model the difference in (excess) mortality before and during the crisis).

### Methodological choices

When fitting an (excess) excess mortality model, several methodological choices have to be made. This section describes the most important choices, including a justification of the choices made for our motivating application.

#### Scale of covariate effects

Firstly, a choice has to be made regarding the effects of covariates on the individual (excess) hazards. The effect of covariates $\textbf{Z}$ on each (excess) hazard can be assumed to be either proportional or additive.

Conventionally, the effect of covariates on the baseline excess hazard $\lambda _{0,excess}(t|\textbf{Z})$ is assumed to be proportional [12, 13]. Hence, the hazard at time *t* for an individual with a *p*-vector of covariates $\textbf{Z} = (Z_1,..,Z_p)$ is given by:

3 $$\begin{aligned} \lambda (t|\textbf{Z}) = \lambda _0(t) \times \exp (\textbf{Z}^T \varvec{\alpha }(t)), \end{aligned}$$

where $\varvec{\alpha }(t)=(\alpha _1(t), \dots , \alpha _p(t))$ denotes the (time-varying) vector of coefficients.

In the additive hazards model, the hazard at time *t* for an individual with a *p*-vector of covariates $\textbf{Z} = (Z_1,..,Z_p)$ is given by:

4 $$\begin{aligned} \lambda (t|\textbf{Z}) = \beta _0(t) + \textbf{Z}^T \varvec{\beta }(t), \end{aligned}$$

with $\varvec{\beta }(t)=(\beta _1(t), \dots , \beta _p(t))$ the vector of coefficients. Hence, at each time point, the hazard is modelled as a linear function of the covariates plus an unspecified baseline hazard. The fact that the baseline hazard is denoted by $\beta _0(t)$ instead of $\lambda _0(t)$ is merely a convention. The version of the model where $\varvec{\beta }(t)$ is allowed to vary over time is known as Aalen’s additive hazards model; the version where all covariate effects are assumed to be constant over time is known as Lin and Ying’s additive hazards model [14].

**Fig. 3:**
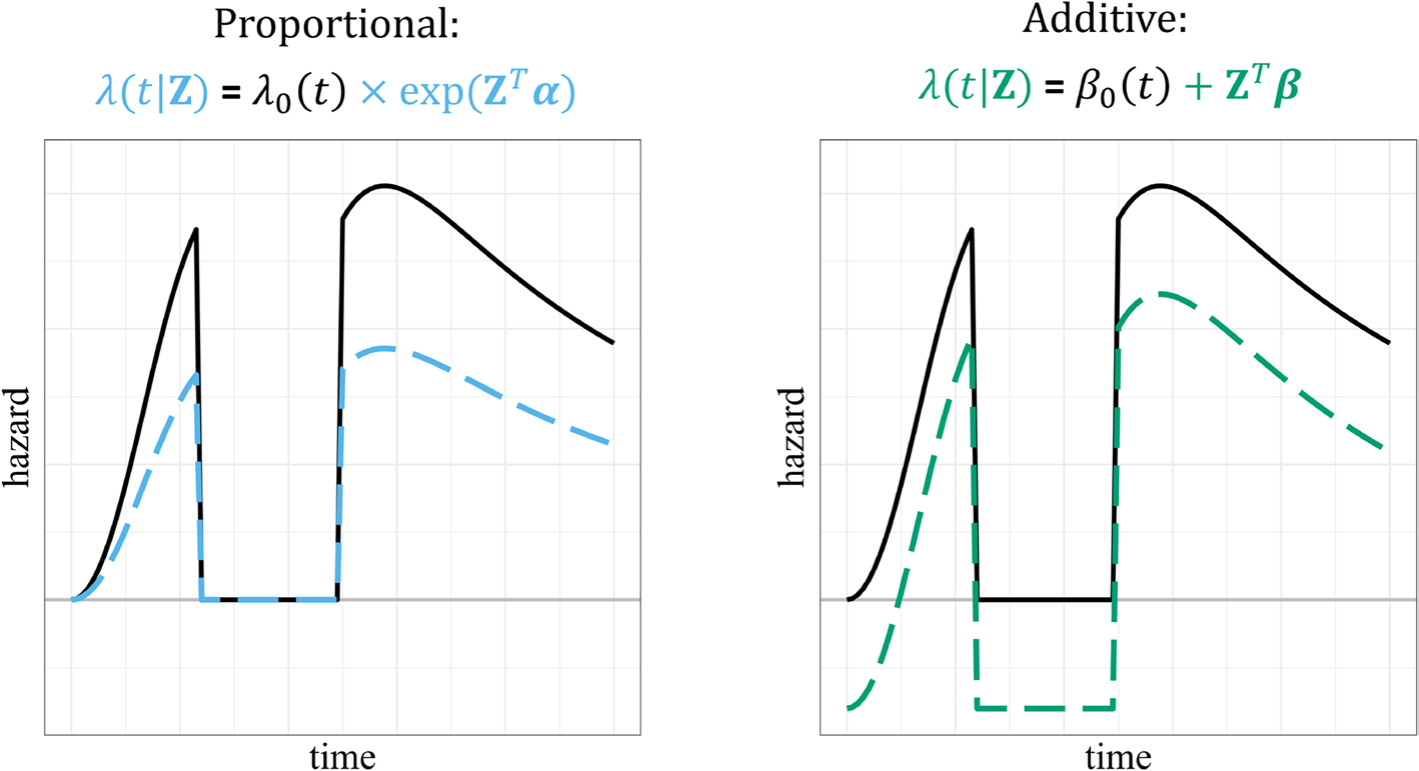
Illustration of the differences between a proportional and an additive hazards model, assuming time-constant effects. The black line, identical in both panels, is a fictional baseline hazard. Included is a small time frame in which this hazard is equal to 0. The colored dashed lines represent the height of the hazard given the linear predictor. In the left panel the effect of the (exponent of the) linear predictor is assumed to be proportional: at every point in time, the dashed blue line is some constant multiple lower than the black line. When the baseline hazard is equal to 0, $\lambda (t|\textbf{Z})$ is as well. In the right panel the effect of the linear predictor is assumed to be additive: at every point in time, the dashed green line is a constant amount lower than the black line. When the baseline hazard is equal to 0, $\lambda (t|\textbf{Z})$ hence is negative

In the context of excess excess mortality models, a useful characteristic of the additive hazards model is that it does not restrict the estimated (excess) hazards to be positive—this in contrast to Cox proportional hazards models, where the Breslow estimator used for the cumulative baseline hazard is restricted to be nonnegative [15]. Since the exponent of the linear predictor is per definition also never negative, the resulting hazard must also be nonnegative in a Cox model. To visualize this, a toy example is provided in Fig. 3, in which for simplicity time-constant effects are assumed for both the proportional and the additive model. In our motivating application, this characteristic of the additive hazards model is particularly relevant, as it is known that the total excess hazard was negative at certain periods in time [3]. This in essence precludes the use of any proportional hazards-based approach. Hence, we used Aalen’s additive hazards model.

Aalen’s additive hazards model is very flexible and relies on few assumptions: besides the common assumption of independent censoring, it assumes a linear effect of continuous covariates at each point in time. Checking the assumption of linearity, necessary for continuous covariates, is often done by visually inspecting plots of the martingale residual processes over time [16, 17] but it is also possible to use log-transformed smoothed pseudo-observations [18]. If the assumption of linearity is not met for a given continuous covariate, this covariate can be transformed or its nonlinear effect can be modelled using splines.

#### Source of background mortality

A second choice in (excess) excess hazard models concerns the source of the background mortality hazard, $\lambda _{background}(t|\textbf{D})$. As mentioned in “Relative survival models” section, the standard approach of using population life tables for this hazard is only justifiable if it is reasonable to assume that the excess hazard of the group under study contributes little to the overall hazard of the general population. A concrete example of a scenario in which the use of population life tables can be justified and one in which it cannot is the following: methods for modeling relative survival are often used in cancer registries, to measure and analyze the survival of those diagnosed with cancer over time. It is generally reasonable to use the overall population life tables for every *individual* cancer type, as removing this particular group of cancer patients from the population would not have a large effect on the hazards in the resulting life tables. However, the excess mortality of all cancer patients *together* does have a significant effect on the observed survival of the general population, as this is a rather large group [19–21]. In such a case using population life tables for $\lambda _{background}(t|\textbf{D})$ cannot be justified.

Also in our motivating application of care home residents during the Covid-19 pandemic, population life tables cannot be used. Care home residents are much more frail than age- and sex-matched peers who are not in a care home, and a considerable percentage of the Dutch 70+ population is in a care home [22]. As $\lambda _{background}(t|\textbf{D})$ should in principle correspond to the hazard of those not in a care home outside the Covid-19 pandemic (see Table 1), a life table-based $\lambda _{background}(t|\textbf{D})$ will be too high and therefore the excess hazards too low. Instead, we use individual-level data from the population of interest to manually construct the appropriate background hazards. An additional advantage of using individual-level data is that it allows for the incorporation of more covariates in the background mortality than sex and age. Furthermore, seasonal effects can be considered: especially for older persons, the daily mortality hazards during winter are higher than during the rest of the year. Population life tables only give the average daily hazard in a given year. By manually constructing the background hazard, this seasonal variation can be taken into account. In our application this is relevant, as the Covid-19 pandemic-related excess mortality experienced by the whole population, $\lambda _{cri}(t|\textbf{D})$, is not very large relative to the background mortality $\lambda _{background}(t|\textbf{D})$: as a result, small changes in the background mortality can have a large effect on the resulting pandemic-related excess mortality.

#### Model timescale

The final choice concerns the timescale of the model. Three meaningful timescales in survival analysis are time since diagnosis or start of treatment, age and calendar time. In many relative survival settings, time since diagnosis or treatment will be the natural choice: e.g. when modeling the excess mortality of breast cancer patients, the occurrence of disease-specific mortality is primarily associated with the time since start of diagnosis, making this the logical timescale to use. In our motivating application the dominant timescale is calendar time: the Covid-19 pandemic occurred in different waves (distinct periods of rising and falling cases of Covid-19 infection), hence the number of infections could vary strongly from week to week and from month to month.

By defining *t* as time since start of a year (in days, in continuous time), it is possible to determine all four (excess) hazards of Eq. (2) by fitting a single additive hazards model. In other words: the timescale of our model is calendar time, but there is a clock-reset at the start of each year. In this way, contrasts between years, and between groups, can conveniently be made to derive the different hazards. We are interested in the Covid-19 pandemic years 2020–2021; the reference years are 2015–2019. This means that if a certain individual in the dataset is observed during multiple years, e.g. 2017–2020, this person will get multiple rows in the dataset: at least one for each year under which they were at risk. An additional variable in the dataset denotes which year each row refers to. If this person changes covariate values (e.g. enters a care home or turns a year older) during the year, they also obtain a new row in the dataset, with the appropriate start and end time. Such a data structure is known as a ‘long’ format [23].

Table 2 provides a toy data set structured in this way. Here, year 3 is assumed to be a crisis year, whereas year 1 and 2 are regular years. Which year each row refers to is given by the variable year. The start and stop time variables t_start and t_stop contain at what day in the year someone enters or leaves, respectively (due to changing a covariate value). Hence, these two variables can take a maximum value of 365 (366 in a leap year). t_start and t_stop are given in continuous time, but for simplicity decimal numbers are not shown here. The indicator variables vul and cri denote whether someone belongs to the vulnerable group of interest and whether it is a period of crisis, respectively. The interaction of these two defines the group for which the excess excess hazard can be determined. For simplicity, other covariates are not included in this toy example. The interpretation is as follows: the individual with id=3 entered the data set on day 120 of year 2 (t_start=120 & year=2). Initially, this person did not belong to the vulnerable group of interest (vul=0), but became so on day 232 of year 3, which was a crisis year (vul=1 & t_start=232 & cri=1). This person died soon thereafter (status=1), on day 359 of year 3 (t_stop=359 & year=3).

The indicator variables vul and cri correspond to the questions on the axes of Table 1, and their interaction results in four unique combinations: [0,0], [1,0], [0,1] and [1,1]. A prerequisite for fitting the additive hazards model is that in each point in time, all four combinations are present in the data. This explains why it is not feasible to choose a timescale such as calendar time without the yearly clock-reset on January 1 when fitting a single model, because then per definition, the pre-Covid-19 pandemic years used as the reference years and the Covid-19 pandemic years will not overlap.

**Table 2:** Toy data set structured in a long format, which makes it possible to fit a single additive hazards model to estimate all (excess) hazards. The timescale is time since start of the year (in days). Year 3 is a crisis year. The indicator variables vul and cri denote whether someone belongs to the vulnerable group of interest and whether it is a period of crisis

id	t_start	t_stop	status	vul	cri	year
1	0	365	0	0	0	1
1	0	365	0	0	0	2
1	0	365	0	0	1	3
2	83	365	0	0	1	3
3	120	365	0	0	0	2
3	0	232	0	0	1	3
3	232	359	1	1	1	3
4	0	164	0	0	0	1
4	164	365	0	1	0	1
4	0	111	0	1	0	2
4	111	286	1	0	0	2

## Data and model details

### Data description and preprocessing

We used anonymized, non-public individual-level population data from Statistics Netherlands to determine the different excess hazard components of Dutch care home residents aged 70 and older during the Covid-19 pandemic. These data are linkable microdata about all registered individuals in the Netherlands, which are available to researchers under strict conditions. The data encompass a wide range of variables (demographic, socio-economic, health-related, etc.) collected through various surveys and administrative registers.

Our population of interest concerned all Dutch inhabitants aged 70 and older during the years 2015–2021. We focused on this particular age group because they are more likely to suffer severe consequences from a Covid-19 infection and to be in a care home than younger people. The years 2015–2019 were used as the reference years; the years 2020–2021 were the Covid-19 crisis years. For each year, only those living in the Netherlands on January 1^st^ were included. Emigration during a given year was not considered, because we did not have access to exact immigration and emigration dates. The years 2016 and 2020 are leap years, with 366 days. Hence, for the 366^th^ day, the value of the background hazard and care home excess hazard are based on 2016 only, and the Covid-19 pandemic-related hazard and excess excess hazard are based on 2020 only.

We constructed a long format dataset, as described in “Model timescale” section, by linking different datasets using a unique pseudonymized individual identifier as the key. Details on which datasets were used can be found in the Supplementary Information, Additional File 2, Table S1. Care home residency was determined by checking who in our population of interest made use of care for which the costs are covered under the Dutch Long-Term Care Act (“Wet langdurige zorg”). Only those who receive care at a care provider’s facility (instead of at their private home) were considered to be part of the vulnerable group of interest.

Our final long format dataset consisted of 33,222,385 rows, containing data on 3,385,242 unique individuals, with information on sex (binary variable sex), year (binary variables y20 and y21 for 2020 and 2021, respectively—the other years are the reference years and hence do not require an indicator, as we take the average over these years for the background hazard), care home residency (binary variable care) and age (continuous variable age). The population size by sex, care home residency and year (on January 1^st^) can be found in the Supplementary Information, Additional File 2, Table S2.

All analyses were performed in the statistical programming language R (version 4.4.0). The additive hazards models were fitted using the *aalen* function from the *timereg* package (version 2.0.5).

### Full model structure

A single Aalen’s additive hazards model was fitted with the following covariates, here given per hazard component, where : denotes an interaction. All associated covariate effects are time-varying.

- $\lambda _{background}(t| \textbf{D})$: (intercept) + age + sex + age:sex
- $\lambda _{vul}(t|\textbf{D},\textbf{W})$: care + care:age + care:sex + care:age:sex
- $\lambda _{cri}(t|\textbf{D})$: y20 + y20:age + y20:sex + y20:age:sex + y21 + y21:age + y21:sex + y21:age:sex
- $\lambda _{vul, cri}(t|\textbf{D},\textbf{W})$: care:y20 + care:y20:age + care:y20:sex + care:y20:age:sex + care:y21 + care:y21:age + care:y21:sex + care:y21:age:sex

Each excess hazard component has its own ‘main effect’ or intercept: care for $\lambda _{vul}(t|\textbf{D},\textbf{W})$, y20 and y21 for $\lambda _{cri}(t|\textbf{D})$, care:y20 and care:y21 for $\lambda _{vul, cri}(t|\textbf{D},\textbf{W})$. Each of these main effects has an interaction with age, sex and the interaction between these two, which allows for differences in effect sizes for age between the sexes. A distinction between y20 and y21 in the two crisis-related excess hazard components is necessary to allow for a different excess mortality pattern in 2020 and 2021.

Age is the only continuous covariate in this model, for which the assumption of linearity must hence be checked. It is likely that untransformed age will not meet this assumption since the effect of age on mortality is known not to be linear [24]. An additional complication is that age is included several times, in various interactions. It is possible that the effect of age is not the same for each interaction, in which case a single transformation of age does not suffice. Finding a different optimal transformation for each time that age is included in the above model, using e.g. log-transformed pseudo-observations or martingale process residual plots as described in “Scale of covariate effects” section, is not feasible: such checks of linearity have to be done for each covariate individually, with no guarantee that in the full (multivariable) model the assumption is still met.

A simple but crude solution to this problem is to stratify age in different groups. However, as categorizing continuous variables should generally be avoided, we instead modelled the effect of age using regression splines. Hence, strictly speaking age should be replaced by $f(\texttt {age})$ in the full model structure given above, where *f*() denotes some regression spline. Regression splines can be used to capture flexible shapes of continuous covariates. In this application natural cubic splines were used, which are *B*-splines of degree 3 with the additional constraint that the spline function is linear beyond the boundary knots. For a detailed discussion of spline types and various modeling options, we refer to Perperoglou et al. [25]. To decide where to place the knots, first a model using stratified age groups (70–79, 80–89 and 90+) was fitted and the effect of age on the various hazard components was visually inspected at several points in time. It was decided to model age using a natural cubic spline with two knots, at ages 80 and 90, and two boundary knots, at ages 70 and 100, i.e. four coefficients per spline. This means that the full model has 48 coefficients, which are allowed to vary at every *t*. See the Supplementary Information, Additional File 1 for a detailed illustration of the effect of age on the different hazard components, including a sensitivity check of the knot placement.

Given the flexibility of Aalen’s additive hazards model and the inclusion of interaction effects between sex and the other covariates in each hazard component, fitting the full model is equivalent to fitting a model for men and women separately. Fitting two separate models was done for computational feasibility, given the size of the full dataset.

To obtain confidence intervals, we performed simple bootstrapping by repeatedly sampling with replacement from the original dataset and repeating the model-fitting procedure. The lower and upper bounds of the 95% confidence intervals can subsequently be determined by obtaining the 2.5^th^ and 97.5^th^ percentiles of the bootstrap distribution, respectively [26, 27]. Reported results are based on 500 bootstrap samples.

### Presentation and interpretation

Relative survival models are typically fitted on the hazard scale. This is convenient because in relative survival the background and excess mortality are treated as competing events. On the hazard scale, the hazard of one event does not depend on the hazard of a competing event [28]. However, interpreting (cumulative) hazards is not straightforward: therefore, cumulative incidences (the probability over time of experiencing a certain event) will also be presented in “Results” section. Cumulative incidences do depend on competing events: competing events prevent the occurrence of the event of interest. The Aalen-Johansen estimator was used to calculate cumulative incidences from the cumulative hazards [29]. Note that as the excess hazards are not constrained to take positive values, the corresponding cumulative incidence can be negative as well.

In our motivating application, membership of the vulnerable group of interest (i.e. being a care home resident) is a time-varying covariate: individuals can move to and from a care home, thereby changing groups as defined in Table 1. If someone entered a care home during the year, they were considered part of the care home group from the moment they enter the home. Hence, at each point *t* all hazards were estimated based on current covariate status. From the four estimated hazards, the cumulative incidences for two hypothetical groups can be directly constructed: those who were always in a care home in 2020–2021, and those who were never in a care home in 2020–2021. This is similar to the construction and interpretation of Simon-Makuch survival curves [30].

As we do not have an explicit model for transitions to/from our vulnerable group of interest, we cannot provide cumulative incidences for the entire group of people that were either in a care home, or not in a care home, on January 1, 2020. However, with our model it is possible to calculate cumulative incidences for a specific individual who e.g. entered a care home halfway the year, by considering the relevant hazard components at each point in time: i.e. for this individual, the care home hazard $\lambda _{vul}(t|\textbf{D},\textbf{W})$ only comes into play when they enter the care home. As there are countless possible patterns of entering and leaving a care home, in the next section results are only reported for the (hypothetical) group that stays in a care home throughout the year.

## Results

**Fig. 4:**
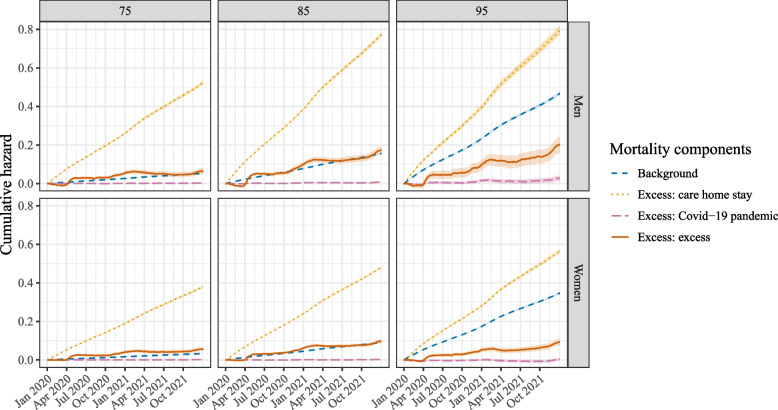
The four different mortality components given for three different ages (75, 85, 95) on the cumulative hazards scale. The shaded areas represent the 95% confidence intervals

Figure 4 contains the four mortality components of the excess excess mortality model as given by Eq. (2), for three different ages (75, 85 and 95) and the two different sexes. Results are presented on the cumulative hazard scale. The figure shows that the blue background hazard, $\lambda _{background}(t|\textbf{D})$, increased with age and was higher for men than for women. The orange line corresponds to the hazard associated with being in a care home, $\lambda _{vul}(t|\textbf{D},\textbf{W})$. It can be seen that this hazard was substantially higher than the background hazard, and also increased with age, although it was about the same size for those aged 85 or 95. The pink Covid-19 pandemic-related excess hazard for non-care home residents, $\lambda _{cri}(t|\textbf{D})$, was small: on the scale of this figure it is very low, except for those aged 95. Finally, the red line represents the excess excess hazard $\lambda _{vul, cri}(t|\textbf{D},\textbf{W})$, the quantity of interest. It can be observed that all groups experienced a positive excess excess cumulative hazard. This hazard was also higher for men than for women and increased with age. It followed a similar pattern as the orange care home residence hazard in that its height is about the same for the oldest two ages shown in the figure. The confidence intervals, based on 500 bootstrap samples, are generally narrow, to the point of not being visible on the scale of this figure. It is widest for the red excess excess mortality component in men aged 95. This makes intuitive sense, since this hazard component is based on the smallest group, and hence most variability is observed here. Additional File 2 of the Supplementary Information contains the cumulative hazards and their 95% confidence intervals on December 31^st^ 2020 (Table S3) and 2021 (Table S4).

**Fig. 5:**
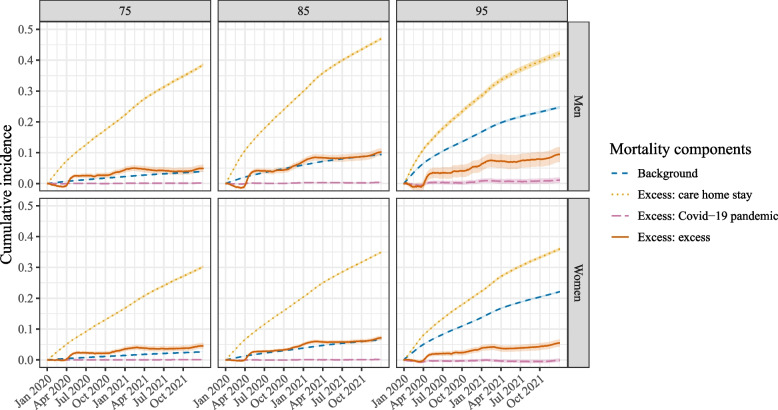
The four different mortality components given for three different ages (75, 85, 95) on the cumulative incidence scale. The shaded areas represent the 95% confidence intervals

In Fig. 5 the results of Fig. 4 are given on the cumulative incidence scale. The two figures show no differences in terms of the relative ordering of the mortality components, but the lines are more curved on the cumulative incidence scale, especially for the older ages. This is due to the fact that over time, an increasing proportion of people will already have died. Even though the individuals remaining at risk have a similar hazard, the increase in cumulative incidence is smaller, as there is a smaller group remaining at risk. The interpretation of the cumulative incidence curves is straightforward: for a woman aged 95 who was in a care home throughout 2020-2021, the cumulative incidence of dying due to the care home stay excess mortality component is 36% at the end of 2021. The red excess excess mortality component was lowest for women aged 75, equalling 4.5% (12.0% of the total observed mortality) at the end of 2021 for those in care home throughout the study period, and highest for men aged 85, equalling 10.3% (15.3% of the total observed mortality).

**Fig. 6:**
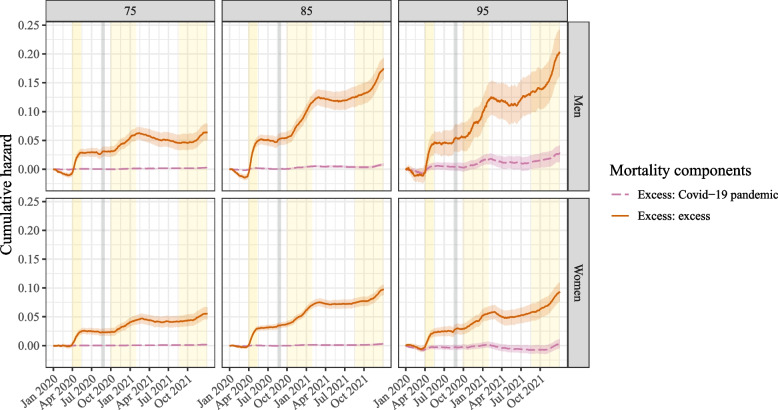
The two crisis-related excess mortality components given for three different ages (75, 85, 95) on the cumulative hazards scale. The yellow shaded areas denote the three Covid-19 waves in the Netherlands; the grey shaded area represents a heat wave. The shaded areas around the cumulative hazards represent the 95% confidence intervals

Figure 6 zooms in on the two hazard components related to the crisis: the Covid-19 pandemic-related hazard and the excess excess hazard. In this figure, the yellow shaded areas represent the three Covid-19-waves experienced in the Netherlands in 2020-2021 [31]. The gray shaded area represents the heat wave that was observed during the summer of 2020, which also resulted in excess mortality. The link between the shape of the cumulative hazard and the Covid-19 waves is clear: during these most severe periods of crisis, the figure shows a sharp increase in the cumulative hazard in all panels. This pattern is visible both for the red excess excess cumulative hazard as well as for the pink Covid-19 pandemic-related excess cumulative hazard (albeit less clearly visible in the latter—on the scale of this figure the pink hazard is still very low for those aged 75). Furthermore, for all ages the red excess excess cumulative hazard stabilized or even dropped from around March 2021 onward, although it started to rise again around September 2021, especially in men.

**Fig. 7:**
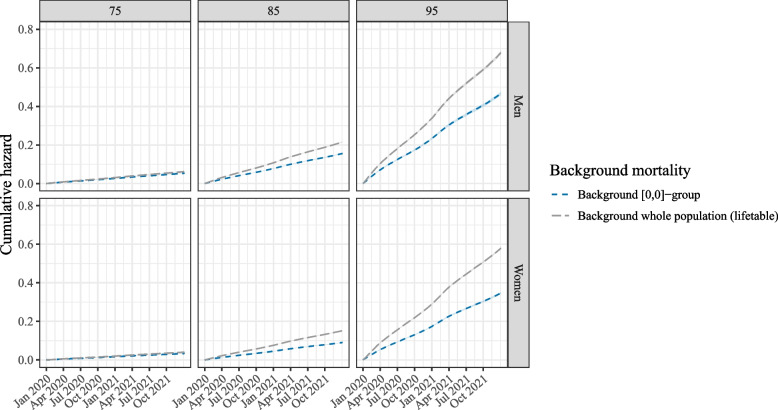
A comparison of two different estimates of the background hazard: the blue line is based on individuals who were not in a care home in 2015–2019 (group [0,0]), the grey line is based on the entire population in 2015–2019. The lines are given for three different ages (75, 85, 95) and are plotted on the cumulative hazards scale. The shaded areas around the cumulative hazards represent the 95% confidence intervals

The importance of choosing the right background or reference population is emphasized by Fig. 7. Here, the blue background hazard used throughout this paper is compared to a background hazard that is based on the entire population in the reference years 2015-2019, i.e. including the care home residents. This is similar to using an external life table for the background hazard, as life tables are also based on the entire population. It can be seen that the higher the age, the larger the divergence between the two lines. This is due to the fact that a higher percentage of people lives in a care home in these older age groups. Of those aged 75, a relatively small percentage lives in a care home, and hence the overall population mortality hazard (in grey) is close to that of those not in a care home (in blue). However, for the other two ages that are shown here the difference is more substantial. A biased background mortality hazard will result in biased estimates of the excess hazards. Hence, this figure serves as a warning to always carefully consider whether it is justified to use whole population life tables to base the background mortality hazard on.

## Discussion

This paper introduced the idea of “excess excess” mortality, which is a useful concept when aiming to disentangle the different excess mortality components of groups experiencing more than one cause of excess mortality, which may interact. As illustrated, this model is useful in the context of vulnerable groups during crises, which are to be compared with a non-vulnerable reference population. Vulnerable groups during crises experience three distinct excess hazards: the excess hazard associated with belonging to the vulnerable group, the excess hazard due to the (direct or indirect) crisis effects that is experienced by the population as a whole, and the excess excess hazard, capturing any remaining excess hazard. This final component provides an answer to the question how much extra risk members of the vulnerable group experienced during the crisis period, compared to those not belonging to this group, taking into account their already heightened risk due to being vulnerable.

In this work all hazard components, including the excess excess hazard, of Dutch care home residents aged 70 and older during the Covid-19 years 2020 and 2021 were quantified by fitting a single model. An additive hazards model was used. Both the overall Covid-19 pandemic excess hazard and the excess excess hazard were negative in the early months of 2020, in line with earlier findings that 2020 started with a relatively ‘mild’ respiratory infection season compared to previous years [32]. At the end of 2021, the excess excess mortality component was lowest for women aged 75 and highest for men aged 85 and 95. In general, men and older age groups experienced higher excess excess mortality. Between March and July of 2021, the cumulative excess excess mortality stabilized or even slightly decreased in all groups. It started to rise again around September 2021, coinciding with the third Covid-19 wave. Vaccination started in January 2021, which might partly explain this pattern. An additional potential explanation for the observed negative excess excess hazard in this period could be that the respiratory infection season was very mild in the winter of 2020–2021, meaning that the incidence of influenza and other respiratory infections was relatively low [33].

Although our study focused on care home residents, the analysis results also reveal interesting insights on community dwellers. Often, excess hazard is reported for an entire population, possibly stratified by age and sex. This is a justified approach when the aim is to obtain an averaged picture of excess mortality. However, the results of this paper show that when modeling the excess hazards specific to care home residents separately, the Covid-19 pandemic-related excess hazard that applies to the entire population is much lower—for the youngest two age groups practically indistinguishable from zero. In other words, when no distinction is made between the excess hazard of those in a care home and those not in a care home, the reported Covid-19 pandemic-related excess hazard will be an average of $\lambda _{cri}(t|\textbf{D})$ and $\lambda _{vul, cri}(t|\textbf{D},\textbf{W})$. This may hide some of the underlying patterns.

Our application differs from conventional relative survival applications in several ways. First and foremost, three excess hazards are included in our model instead of only one. Secondly, comparisons are not only drawn between different groups in the same time period (i.e. care home residents versus community dwellers), but also between the same group in different time periods (i.e. before and during the Covid-19 pandemic), all in the same model. Thirdly, individual-level population data was used to model all hazards, instead of relying on pre-existing life tables for the background hazard. We illustrated that population life tables would have resulted in biased estimates: care home residents are an important part of the 70+ population, with a much higher mortality hazard, and hence have a substantial influence on the life tables. Finally, in our application an additive hazards model was used, instead of the more commonly encountered (Cox) proportional hazards model. This was done because negative excess hazards were observed, which precluded the use of any proportional hazards-based approach. As an additional advantage, by using the additive hazards model all hazard components can be quantified by fitting a single model.

The additive hazards model was a natural choice in our motivating application, but in other contexts proportional hazards models might be more appropriate. We plan to extend the excess excess mortality model framework to also allow for proportional hazards models in future work, although this will likely be much more computationally complex than the additive approach introduced in this paper. Even in the standard relative survival setting with a single excess hazard component, an iterative EM-algorithm is required if the excess hazard is modelled as in a Cox proportional hazards model (i.e. with an unspecified baseline hazard and a proportional effect of the linear predictor on this hazard) [13]. The additive approach, on the other hand, has a closed-form expression for the estimates and does not require an iterative approach. Note that the term ‘proportional’ refers to the effect of the covariates on the different baseline hazards, not to the relation between the background hazard and excess hazard(s): this remains additive. Models where the effect of the excess hazard on the background hazard is multiplicative exist but have a less obvious interpretation and are considered less realistic in most settings [34].

We used regression splines to capture the nonlinear effect of age. This required a manual specification of the number and location of the knots. Given the size of our dataset and the fact that the effect of age was found to be (approximately) monotone over time, this manual choice was not considered to be a problem. This was confirmed by the additional analyses that we performed, which showed that the number and location of the knots hardly mattered in terms of the resulting hazard component estimates (Supplementary Information, Additional File 1). However, in smaller-scale applications, it might be preferable to consider penalized B-splines [35], where the number and location of the knots are automatically determined.

Care home residency is a time-varying covariate in our model: people are allowed to change group membership over time. As discussed in “Presentation and interpretation” section, we therefore presented our results for the hypothetical group of individuals who stayed in a care home throughout 2020-2021. To explicitly model the individual paths in and out of the care home, an extension of our model towards a multi-state model would be required [23]. A multi-state model would allow for a comparison of predictions for those who were in a care home on January 1^st^, 2020 versus those who were not.

A limitation of our model is the need for detailed data on the vulnerable group of interest. Adjustment for relevant covariates is essential to meet the relative survival assumption that the vulnerable group of interest is similar to some reference population (i.e. shares the same background hazard) except for the characteristic defining its vulnerability. Translated to our model, an implicit assumption is that, conditional on age and sex, the background characteristics of care home residents during 2020–2021 were the same as in 2015–2019. This might not hold and the direction of the bias could go both ways. It is possible that more people, in particular those less frail who were still capable of taking care of themselves with (extra) help from informal caregivers, decided to keep living at home in 2020–2021, even though a place in the care home would have been available for them. It is also possible that, given the increased mortality rates in care homes in 2020–2021, more places than usual became available, which could mean that for individuals who would not have been considered frail enough for admission before 2020–2021, places did become available. In principle, a potential difference in background characteristics of care home residents can be accounted for by including relevant covariates in the excess excess mortality model. We did not do so in this paper, as our main aim was the introduction of this model in general. Hence we did not introduce too much complexity to the model to analyze the motivating data example.

It should be noted that relative survival is not the only approach to estimate excess mortality. Depending on the context, it might be preferable to quantify excess mortality using methods that do not rely on the relative survival assumption of a shared background hazard and do not require detailed covariate information. For example, when comparing excess mortality during the Covid-19 pandemic between different countries, a popular alternative metric is the so-called “P-score”: the ratio of the excess deaths to the expected deaths, expressed as a percentage [36, 37]. The P-score can be used to compare countries. Another popular approach is to fit a (quasi) Poisson model, directly modeling the absolute number of excess deaths [38]. These metrics might be better suited to the situation where the aim is to compare separate populations and/or when there is only one cause of excess hazard. This is different from the scenario that we considered in this paper, in which the vulnerable group of interest experiences two individual causes of excess mortality, which may interact, and shares the same background hazard with the non-vulnerable general population.

Our real data results are primarily descriptive and should be interpreted with caution, avoiding causal claims. That is, our results should not be taken as an indication that it would have been better to have taken family members or friends out of the care home during the pandemic. Care home residents are frail, more so than age- and sex-matched community dwellers, so it is no surprise that these residents experienced a certain degree of excess excess hazard. Our aim was to quantify the extent to which this occurred. At the same time, it should not be ignored that the excess excess mortality was substantial, in particular in the oldest age groups. Whether our results justify more stringent protective measures in care homes during a future pandemic remains an open question. For example, the social distancing measures that were in place likely negatively impacted the well-being of older people [39]. Whether more stringent measures in care homes would be worth the additional toll on mental health in a future pandemic is a question for policy makers, clinicians and care home residents and their relatives.

We introduced the excess excess mortality model in the context of vulnerable groups during a crisis. Hence, we expected all excess hazards to be positive, at least most of the time. However, as mentioned, strictly speaking excess hazards are not real hazards but differences between real hazards, and hence may be negative. One could therefore also use the excess excess model in different settings, e.g. for groups that are not more vulnerable, but more resilient than the general population. Of the three excess hazards, $\lambda _{cri}(t|\textbf{Z})$ is likely always positive: it is difficult to imagine an ‘anti-crisis’ that is temporary and affects the entire population. For $\lambda _{vul}(t|\textbf{Z})$ and $\lambda _{vul, cri}(t|\textbf{Z})$ it is possible to think of scenarios in which each could be either negative or positive. Note that if $\lambda _{vul}(t|\textbf{Z})$ is negative, the group of interest is actually not more vulnerable, but more resilient than the general population. For simplicity, we stick with the subscript *vul* here. For example, clinicians in the Netherlands have a negative $\lambda _{vul}(t|\textbf{Z})$, as life expectancy has a strong link with socioeconomic position [40]. However, during the Covid-19 pandemic, the direction of the excess excess hazard for clinicians $\lambda _{vul, cri}(t|\textbf{Z})$ is unknown: it might have been positive, as they may have had more interaction with infected persons during the Covid-19 pandemic. Yet, it could also have been negative, as clinicians might have been better aware than the general population how important it was to adhere to the anti-Covid-19 measures and were prioritized during the vaccination campaigns. Hence, the excess excess mortality model presented in this paper can be used in more settings than only that of vulnerable groups during a crisis: in its broadest sense, it can be used to disentangle the excess mortality components of any group experiencing more than one cause of excess mortality.

## Conclusion

The concept of excess excess mortality provides a powerful framework for disentangling multiple components of excess mortality, particularly in the context of vulnerable groups during crises. We demonstrated the utility of this approach using data on Dutch care home residents aged 70 and older during the Covid-19 pandemic. Our findings underscore the importance of distinguishing between population-wide crisis effects and group-specific vulnerabilities. The use of additive hazard models allowed for direct estimation of these components while allowing for negative excess hazards.

The flexibility of the excess excess mortality model makes it applicable to a wide range of settings, including resilient groups or scenarios beyond crisis contexts. Ultimately, this work provides a foundation for more nuanced analyses of excess mortality in scenarios where there is more than one cause or indicator of excess mortality.

## Supplementary information

Supplementary Material 1.

Supplementary Material 2.

Supplementary Material 3.

## Data Availability

Results are based on calculations by the authors using non-public microdata from Statistics Netherlands. Under certain conditions, these microdata are accessible for statistical and scientific research. For further information: microdata@cbs.nl. All analyses were conducted in the statistical programming language R. The code for the main analyses can be found in the Supplementary Information, Additional File 3.

## References

[1] Nordentoft M, Wahlbeck K, Hällgren J, Westman J, Ösby U, Alinaghizadeh H, et al. Excess mortality, causes of death and life expectancy in 270,770 patients with recent onset of mental disorders in Denmark, Finland and Sweden. PLoS ONE. 2013;8(1):e55176. 23372832 10.1371/journal.pone.0055176 PMC3555866

[2] Shah SM, Carey IM, Harris T, DeWilde S, Cook DG. Mortality in older care home residents in England and Wales. Age Ageing. 2013;42(2):209–15. 23305759 10.1093/ageing/afs174

[3] Wang H, Paulson KR, Pease SA, Watson S, Comfort H, Zheng P, et al. Estimating excess mortality due to the COVID-19 pandemic: a systematic analysis of COVID-19-related mortality, 2020–21. Lancet. 2022;399(10334):1513–36. 35279232 10.1016/S0140-6736(21)02796-3 PMC8912932

[4] Rijpma A, Van Dijk IK, Schalk R, Zijdeman RL, Mourits RJ. Unequal excess mortality during the Spanish Flu pandemic in the Netherlands. Econ Hum Biol. 2022;47:101179. 36399930 10.1016/j.ehb.2022.101179 PMC9468303

[5] Ekamper P, Bijwaard G, van Poppel F, Lumey L. War-related excess mortality in the Netherlands, 1944–45: new estimates of famine-and non-famine-related deaths from national death records. Hist Methods J Quant Interdisc Hist. 2017;50(2):113–28. 10.1080/01615440.2017.1285260 PMC6226247 30416230

[6] Bär M, Bom JA, Bakx PL, Hertogh CM, Wouterse B. Variation in Excess Mortality Across Nursing Homes in the Netherlands During the COVID-19 Pandemic. J Am Med Dir Assoc. 2024;25(9):105116. 38950583 10.1016/j.jamda.2024.105116

[7] Sepulveda ER, Stall NM, Sinha SK. A comparison of COVID-19 mortality rates among long-term care residents in 12 OECD countries. J Am Med Dir Assoc. 2020;21(11):1572–4. 33138940 10.1016/j.jamda.2020.08.039 PMC7486852

[8] Gadeyne S, Rodriguez-Loureiro L, Surkyn J, Van Hemelrijck W, Nusselder W, Lusyne P, et al. Are we really all in this together? The social patterning of mortality during the first wave of the COVID-19 pandemic in Belgium. Int J Equity Health. 2021;20(1):258. 34922557 10.1186/s12939-021-01594-0 PMC8684273

[9] Modig K, Lambe M, Ahlbom A, Ebeling M. Excess mortality for men and women above age 70 according to level of care during the first wave of COVID-19 pandemic in Sweden: a population-based study. Lancet Reg Health–Eur. 2021;4;100072. 10.1016/j.lanepe.2021.100072 PMC8454796 34557812

[10] Comas-Herrera A, Patel J, Arling G, Mossong J, Schmidt A. International data on deaths attributed to COVID-19 among people living in care homes. The LTCcovid International Living report; 2022.

[11] Pohar M, Stare J. Making relative survival analysis relatively easy. Comput Biol Med. 2007;37(12):1741–9. 17582396 10.1016/j.compbiomed.2007.04.010

[12] Remontet L, Bossard N, Belot A, Esteve J, of Cancer Registries FRANCIM FN. An overall strategy based on regression models to estimate relative survival and model the effects of prognostic factors in cancer survival studies. Stat Med. 2007;26(10):2214–28. 10.1002/sim.2656 16900570

[13] Pohar Perme M, Henderson R, Stare J. An approach to estimation in relative survival regression. Biostatistics. 2009;10(1):136–46. 18599516 10.1093/biostatistics/kxn021

[14] Lin DY, Ying Z. Semiparametric analysis of the additive risk model. Biometrika. 1994;81(1):61–71.

[15] Lin D. On the Breslow estimator. Lifetime Data Anal. 2007;13:471–80. 17768681 10.1007/s10985-007-9048-y

[16] Aalen OO. Further results on the non-parametric linear regression model in survival analysis. Stat Med. 1993;12(17):1569–88. 8235179 10.1002/sim.4780121705

[17] Lefebvre F, Giorgi R. A strategy for optimal fitting of multiplicative and additive hazards regression models. BMC Med Res Methodol. 2021;21(1):100. 33957858 10.1186/s12874-021-01273-2 PMC8101173

[18] Pohar Perme M, Andersen PK. Checking hazard regression models using pseudo-observations. Stat Med. 2008;27(25):5309–28. 18712781 10.1002/sim.3401 PMC2749183

[19] Ederer F. The relative survival rate: a statistical methodology. Natl Cancer Inst Monogr. 1961;6:101–21. 13889176

[20] Talbäck M, Dickman PW. Estimating expected survival probabilities for relative survival analysis-exploring the impact of including cancer patient mortality in the calculations. Eur J Cancer. 2011;47(17):2626–32. 21924892 10.1016/j.ejca.2011.08.010

[21] Hinchliffe SR, Dickman PW, Lambert PC. Adjusting for the proportion of cancer deaths in the general population when using relative survival: a sensitivity analysis. Cancer Epidemiol. 2012;36(2):148–52. 22000329 10.1016/j.canep.2011.09.007

[22] D VO, Koper I. Het leven in een verpleeghuis: landelijk overzicht van de leefsituatie, ervaren kwaliteit van leven en zorg van oudere verpleeghuisbewoners in 2019. The Netherlands Institute for Social Research (SCP); 2021.

[23] Putter H, Fiocco M, Geskus RB. Tutorial in biostatistics: competing risks and multi-state models. Stat Med. 2007;26(11):2389–430. 17031868 10.1002/sim.2712

[24] Bongaarts J. Long-range trends in adult mortality: models and projection methods. Demography. 2005;42(1):23–49. 15782894 10.1353/dem.2005.0003

[25] Perperoglou A, Sauerbrei W, Abrahamowicz M, Schmid M. A review of spline function procedures in R. BMC Med Res Methodol. 2019;19:1–16. 30841848 10.1186/s12874-019-0666-3 PMC6402144

[26] Efron B. Bootstrap methods: another look at the jackknife. In: Annuals of Statistics, vol. 7. Springer; 1979. pp. 1–26.

[27] Efron B, Tibshirani R. Bootstrap methods for standard errors, confidence intervals, and other measures of statistical accuracy. Stat Sci. 1986;1:54–75.

[28] Andersen PK, Geskus RB, de Witte T, Putter H. Competing risks in epidemiology: possibilities and pitfalls. Int J Epidemiol. 2012;41(3):861–70. 22253319 10.1093/ije/dyr213 PMC3396320

[29] Aalen OO, Johansen S. An empirical transition matrix for non-homogeneous Markov chains based on censored observations. Scand J Stat. 1978:141–50.

[30] Simon R, Makuch RW. A non-parametric graphical representation of the relationship between survival and the occurrence of an event: application to responder versus non-responder bias. Stat Med. 1984;3(1):35–44. 6729287 10.1002/sim.4780030106

[31] RIVM, CBS. Sterfte en oversterfte in 2020 en 2021. National Institute for Public Health and the Environment (RIVM), Statistics Netherlands (CBS)); 2022.

[32] Reukers DFM, van Asten L, Brandsema PS, Dijkstra F, Hendriksen JMT, van der Hoek W, et al. Annual report Surveillance of influenza and other respiratory infections in the Netherlands: winter 2019/2020. National Institute for Public Health and the Environment (RIVM); 2021.

[33] Reukers DFM, van Asten L, Brandsema PS, Dijkstra F, Hendriksen JMT, Hooiveld M, et al. Annual report Surveillance of COVID-19, influenza and other respiratory infections in the Netherlands: winter 2020/2021. National Institute for Public Health and the Environment (RIVM); 2021.

[34] Dickman PW, Sloggett A, Hills M, Hakulinen T. Regression models for relative survival. Stat Med. 2004;23(1):51–64. 14695639 10.1002/sim.1597

[35] Eilers PH, Marx BD. Flexible smoothing with B-splines and penalties. Stat Sci. 1996;11(2):89–121.

[36] Msemburi W, Karlinsky A, Knutson V, Aleshin-Guendel S, Chatterji S, Wakefield J. The WHO estimates of excess mortality associated with the COVID-19 pandemic. Nature. 2023;613(7942):130–7. 36517599 10.1038/s41586-022-05522-2 PMC9812776

[37] Mostert S, Hoogland M, Huibers M, Kaspers G. Excess mortality across countries in the Western World since the COVID-19 pandemic: ‘Our World in Data’estimates of January 2020 to December 2022. BMJ Public Health. 2024;2(1). 10.1136/bmjph-2023-000282 PMC13482250 42614536

[38] Barnard S, Chiavenna C, Fox S, Charlett A, Waller Z, Andrews N, et al. Methods for modelling excess mortality across England during the COVID-19 pandemic. Stat Methods Med Res. 2022;31(9):1790–802. 34693801 10.1177/09622802211046384 PMC9465060

[39] Li L, Taeihagh A, Tan SY. A scoping review of the impacts of COVID-19 physical distancing measures on vulnerable population groups. Nat Commun. 2023;14(1):599. 36737447 10.1038/s41467-023-36267-9 PMC9897623

[40] Kalwij AS, Alessie RJ, Knoef MG. The association between individual income and remaining life expectancy at the age of 65 in the Netherlands. Demography. 2013;50(1):181–206. 22975777 10.1007/s13524-012-0139-3

