# Supplementary material for: A new framework for disentangling different components of excess mortality applied to Dutch care home residents during Covid-19
Source: BMC Med Res Methodol. 2025 May 10;25:126. doi: 10.1186/s12874-025-02579-1 (PMC12065346; doi:10.1186/s12874-025-02579-1)
Supplement: Supplementary file 1 — Supplementary Material 1. [file 12874_2025_2579_MOESM1_ESM.pdf]

Supplementary Information: Additional File 1

A new framework for disentangling different components of excess mortality applied to Dutch care home residents during Covid-19

Marije H. Sluiskes[1] (m.h.sluiskes@lumc.nl), Eva A.S. Koster[1], Jelle J. Goeman[1], Mar Rodríguez-Girondo[1], Hein Putter[1,2], Liesbeth C. de Wreede[1] (l.c.de_wreede@lumc.nl)

[1] *Medical Statistics, Biomedical Data Sciences, Leiden University Medical Center, Leiden, The Netherlands*

[2] *Mathematical Institute, Leiden University, Leiden, The Netherlands*

This document describes the steps that were taken to model the continuous covariate age using a natural cubic spline, when fitting the excess excess mortality model as given by Equation 2 of the main manuscript.

Section 3.2 of the main manuscript contains an overview of the covariates that were included in the model. Age is the only continuous covariate. Its effect is known not to be linear, so we used regression splines to model its effect. This requires a manual specification of the number of knots and the knot placement position.

In our final model, we modelled age using a natural cubic spline with two knots, at ages 80 and 90, and two boundary knots, at ages 70 and 100. This document describes the choices that were made and the sensitivity checks that were performed.

As a first step, we fitted a model where age was split up in three different categories: 70-79, 80-89 and 90+. Results are provided in Figure S1 below.

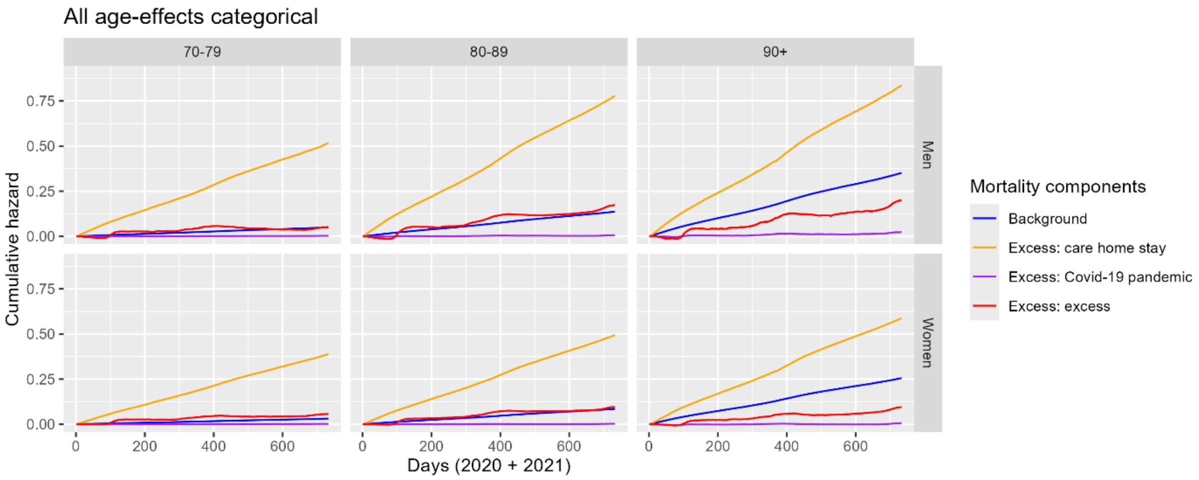

Figure S1. The four different mortality components plotted on the cumulative hazards scale for the three different age groups. Age is modelled as a categorical variable.

Categorizing continuous variables should generally be avoided. In this model, it restricts everyone in the same age group to have the same effect sizes. Therefore, we instead fitted a model where age is modelled using a natural cubic spline. Cubic splines are splines of degree 3; natural splines restrict the spline function to be linear outside the boundary knots, as polynomial splines can be erratic at the boundaries of the data [1]. The boundary knots were therefore placed at ages 70 (the lowest age we consider) and 100 (although there are some people older than 100, the data is very sparse here). As a first attempt, we chose evenly spaced knots, i.e. at ages 80 and 90. This coincides with the age groups as given in Figure S1.

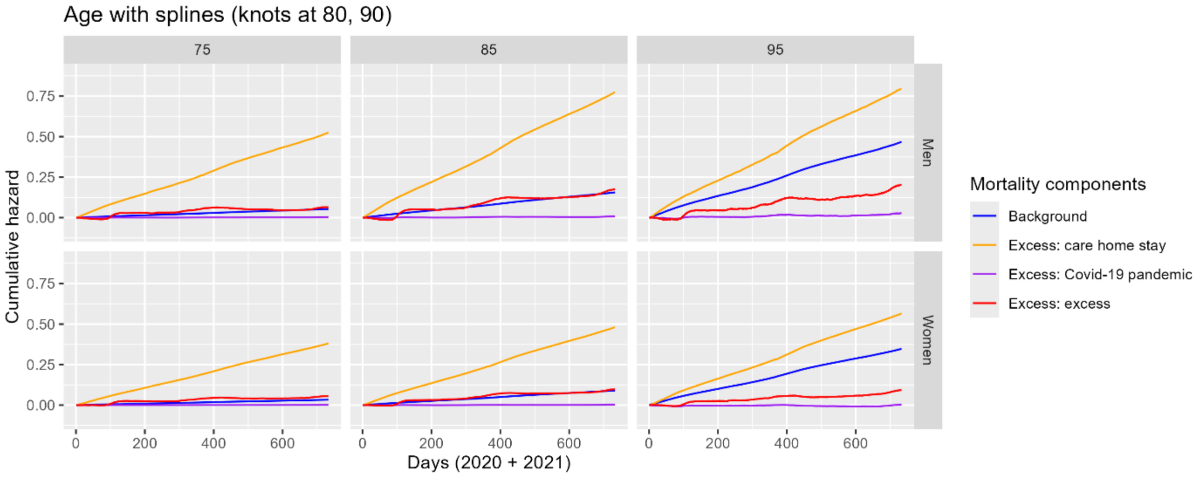

Figure S2. The four different mortality components plotted on the cumulative hazards scale for reference persons with different ages. Age is modelled using a natural cubic spline, with knots at ages 80 and 90.

It can be seen that, in terms of the height of the lines, the result do not differ by much between Figures S1 and S2: the biggest differences are to be found in the 95+ panel, but this is likely due to the fact that we are now no longer plotting results for an entire age group (90+) but for a specific age (95).

Instead of having the number of days in 2020 and 2021 on the x-axis, we can also take a cross-section at a given day, and plot age on the x-axis. This is shown in Figure S3 below, for day 300 (in both 2020 and 2021: the yellow and blue lines are the same, regardless of year, and for the purple and red line there are now two lines).

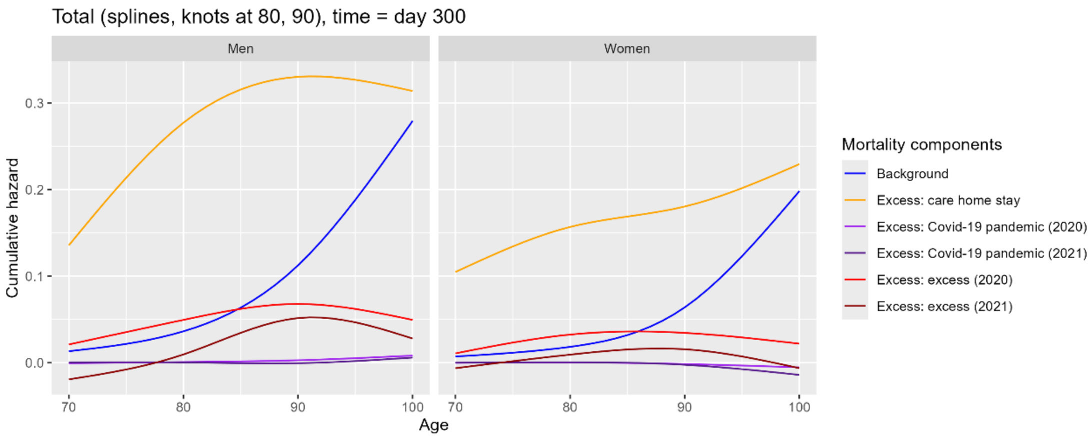

Figure S3. The four different mortality components plotted on the cumulative hazards scale at day 300 (of 2020 and 2021), provided for all ages. Age is modelled using a natural cubic spline, with knots at ages 80 and 90.

Figure S3 can be interpreted as follows: for men aged 85, the yellow cumulative hazard related to care home stay was around 0.32 on day 300 of 2020. Figure 2 shows that this was indeed the case.

As a next step, all cumulative hazards can be shifted vertically such that they are equal to 0 at age 70. In essence, this means removing all age-independent effects, so that the shape of the lines can be investigated and compared between different models. Results are shown in Figure S4. Clearly, the shape of the yellow cumulative hazard is different

from that of the blue one. This suggests that the effect of age differs per hazard component.

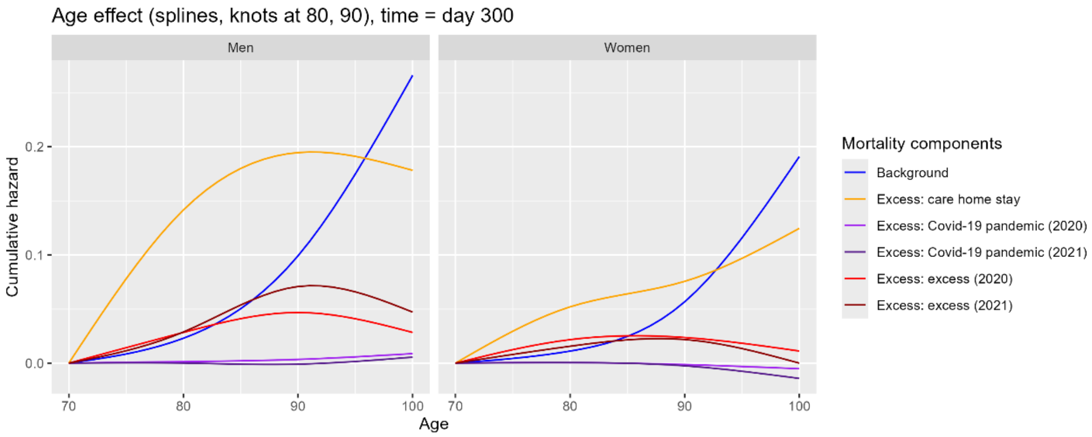

Figure S4. The four different mortality components plotted on the cumulative hazards scale at day 300 (of 2020 and 2021), provided for all ages and centered at 0 for age 70. Age is modelled using a natural cubic spline, with knots at ages 80 and 90.

Eventually, the simplest model that still accurately reflects the data should be selected. Therefore, as a next step we fitted a model with only one knot (keeping the same boundary knots), to see to what extent results change. If they hardly change, the model with one knot for age is to be preferred over the (more complex) model with two knots. As there appears to be an inflection point around age 85 in Figure S4, we fitted the same model but modelling age with a single knot, at age 85. Results of this model are provided in Figure S5.

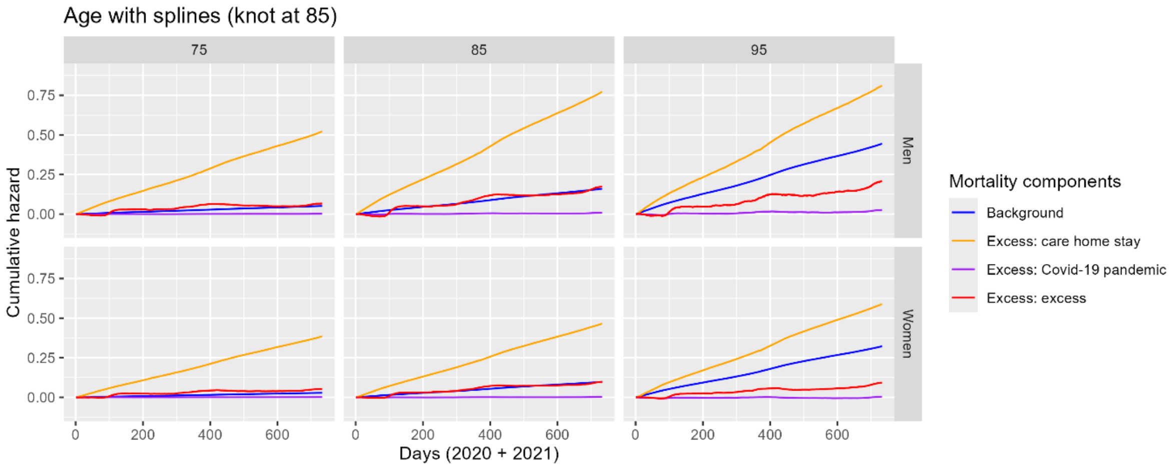

Figure S5. The four different mortality components plotted on the cumulative hazards scale for reference persons with different ages. Age is modelled using a natural cubic spline, with a knot at age 85.

When comparing Figures S5 and S2, some subtle differences are visible: the yellow and blue lines are further apart when only using 1 knot, especially for those aged 95. This suggests that a single knot might be too restrictive. This is confirmed by Figure S6 below: whereas the blue line tended to follow an almost exponential pattern in Figure S4 (which is also in line with what we know about the effect of age on overall mortality rates), the slope is less steep now.

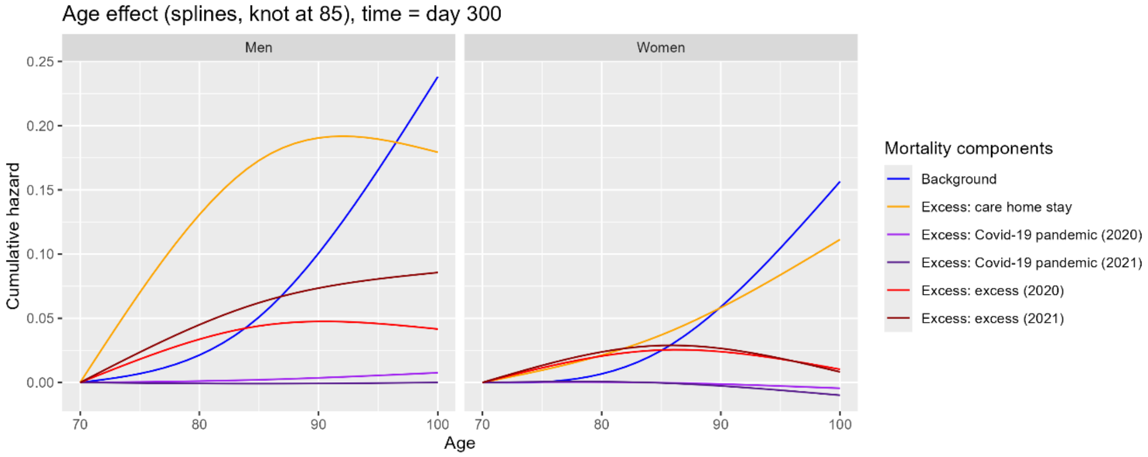

Figure S6. The four different mortality components plotted on the cumulative hazards scale at day 300 (of 2020 and 2021), provided for all ages and centered at 0 for age 70. Age is modelled using a natural cubic spline, with a knot at age 85.

Although the differences between the models with one or two knots are not very large, we preferred the model with two knots, as it captured the exponential effect of age on (background) mortality better.

As a next step, we also investigated a model with three knots, at ages 75, 85 and 95, to check if a model with 2 knots is perhaps still too restrictive. Results are provided in Figure S7.

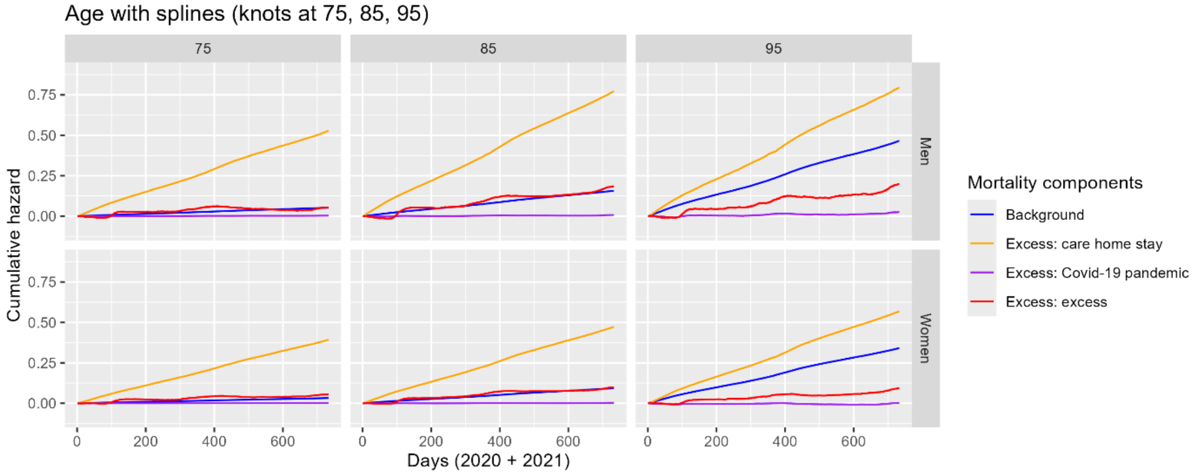

Figure S7. The four different mortality components plotted on the cumulative hazards scale for reference persons with different ages. Age is modelled using a natural cubic spline, with knots at ages 75, 85 and 95.

Figures S7 and S2 are virtually identical. Figures S8 and S4 are very similar as well, although Figure S8 appears to be a bit wiggly (possibly indicating too much flexibility of the model). Apparently, the additional flexibility of a third knot is not needed. Hence, we stick with the simpler model: one with two knots, at ages 80 and 90.

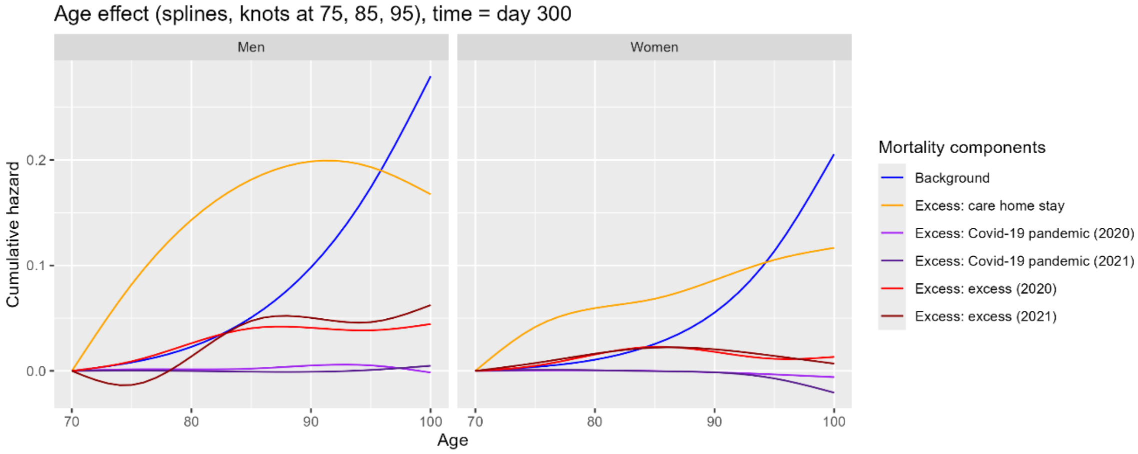

Figure S8. The four different mortality components plotted on the cumulative hazards scale at day 300 (of 2020 and 2021), provided for all ages and centered at 0 for age 70. Age is modelled using a natural cubic spline, with knots at ages 75, 85 and 95.

Note that this application is relatively simple, given that the number and location of the knots did not matter much in terms of the resulting estimates of the cumulative hazard components. Even when fitting age as a categorized variable, the cumulative hazard estimates did not change much, as shown in Figure S1. This might not always hold in other scenarios, in which case the knot placement choice will be more delicate. In such cases one might consider using penalized B-splines instead [2], where the number and location of the knots are automatically determined.

References

[1] Perperoglou A, Sauerbrei W, Abrahamowicz M, Schmid M. A review of spline function procedures in R. BMC Medical Research Methodology. 2019;19:1–16.

[2] Eilers PH, Marx BD. Flexible smoothing with B-splines and penalties. Statistical Science. 1996;11(2):89–121.
