# Supplementary material for: A new framework for disentangling different components of excess mortality applied to Dutch care home residents during Covid-19
Source: BMC Med Res Methodol. 2025 May 10;25:126. doi: 10.1186/s12874-025-02579-1 (PMC12065346; doi:10.1186/s12874-025-02579-1)
Supplement: Supplementary file 2 — Supplementary Material 2. [file 12874_2025_2579_MOESM2_ESM.pdf]

Supplementary Information: Additional File 2

A new framework for disentangling different components of excess mortality applied to Dutch care home residents during Covid-19

Marije H. Sluiskes[1] (m.h.sluiskes@lumc.nl), Eva A.S. Koster[1], Jelle J. Goeman[1], Mar Rodríguez-Girondo[1], Hein Putter[1,2], Liesbeth C. de Wreede[1](l.c.de_wreede@lumc.nl)
[1] *Medical Statistics, Biomedical Data Sciences, Leiden University Medical Center, Leiden, The Netherlands*
[2] *Mathematical Institute, Leiden University, Leiden, The Netherlands*

Table S1. Overview of the used microdata tables and variables.

Datasets	Variables	Original variables used for identification	Comment
**All datasets**	Personal identifier	RINPERSOON	Used to link different datasets
**GBASTAND-BEVOLKINGTAB**	Zip code	GBAPOSTCODE4GAAF 1JANUARI	Used to identify whether person was living in the Netherlands at 1 January of each year
**GBAPERSOONTAB**	Age (rounded down to nearest integer)	GBAGEBOORTEDAG, GBAGEBOORTEMAAN D and GBAGEBOORTEJAAR	Birthdays in microdata of Statistics Netherlands are always set to the first of the month
	Sex	GBAGESLACHT	
**GBAOVERLIJDENTA B**	Date of death	GBADatumOverlijden	
**GEBWLZTAB**	Start date of using care from the Wlz (Long Term Care Act),<br>End date of using care from the Wlz,<br>Care home residency	BEGINWLZGEB, EINDEWLZGEB and WLZLEVERINGSVOR M	WLZLEVERINGSVORM contains the type of care received (e.g. in a care home or at home).

Table S2. Total population size by sex, care home residency and year (on January 1st).

Year	Sex	In care home	Population size
2015	Women	No	1037979
2015	Women	Yes	102623
2015	Men	No	829684
2015	Men	Yes	37091
2016	Women	No	1066187
2016	Women	Yes	92691
2016	Men	No	858839
2016	Men	Yes	34315
2017	Women	No	1121255
2017	Women	Yes	89512
2017	Men	No	917634
2017	Men	Yes	34058
2018	Women	No	1166828
2018	Women	Yes	87647
2018	Men	No	967713
2018	Men	Yes	34671
2019	Women	No	1204155
2019	Women	Yes	87070
2019	Men	No	1010574
2019	Men	Yes	36128
2020	Women	No	1238890
2020	Women	Yes	87364
2020	Men	No	1049753
2020	Men	Yes	37220
2021	Women	No	1267412
2021	Women	Yes	85608
2021	Men	No	1080365
2021	Men	Yes	36563

Table S3. Cumulative hazards on December 31st, 2020. The starting point is January 1st, 2020. The 95% confidence intervals, based on 500 nonparametric bootstrap samples, are provided in the square brackets.

Age	Sex	Background	Ex: ch stay	Ex: Covid-19	Ex: excess
75	Men	0.0266 [0.0263-0.0268]	0.2620 [0.2581-0.2661]	0.0011 [0.005-0.0016]	0.0555 [0.0452-0.0654]
85	Men	0.0779 [0.0770-0.0785]	0.3865 [0.3815-0.3913]	0.0040 [0.0025-0.0057]	0.1018 [0.0884-0.1127]
95	Men	0.2333 [0.2291-0.2371]	0.3974 [0.3859-0.4080]	0.0147 [0.0065-0.0232]	0.0987 [0.0733-0.1252]
75	Women	0.0166 [0.0164-0.0168]	0.1902 [0.1872-0.1928]	0.0005 [0.0001-0.0009]	0.0406 [0.0335-0.0488]
85	Women	0.0452 [0.0448-0.0457]	0.2400 [0.2377-0.2421]	0.0011 [0.0000-0.0022]	0.0617 [0.0558-0.0677]
95	Women	0.1734 [0.1712-0.1755]	0.2823 [0.2777-0.2867]	0.0005 [-0.0041-0.0057]	0.0528 [0.0422-0.0633]

Ex: ch stay = excess hazard component due to being a care home resident, Ex: Covid-19 = excess hazard component due to the Covid-19 pandemic experienced by the entire population, Ex: excess = excess excess hazard.

Table S4. Cumulative hazards on December 31st, 2021. The starting point is January 1st, 2020. The 95% confidence intervals, based on 500 nonparametric bootstrap samples, are provided in the square brackets.

Age	Sex	Background	Ex: ch stay	Ex: Covid-19	Ex: excess
75	Men	0.0532 [0.0527-0.0536]	0.5238 [0.5166-0.5327]	0.0027 [0.0018-0.0035]	0.0644 [0.0501-0.0790]
85	Men	0.1555 [0.1541-0.1570]	0.7726 [0.7634-0.7831]	0.0077 [0.0054-0.0101]	0.1751 [0.1569-0.1934]
95	Men	0.4664 [0.4585-0.4746]	0.7947 [0.7716-0.8163]	0.0274 [0.0131-0.0415]	0.2015 [0.1606-0.2416]
75	Women	0.0331 [0.0328-0.0335]	0.3804 [0.3746-0.3857]	0.0018 [0.0012-0.0024]	0.0551 [0.0456-0.0668]
85	Women	0.0904 [0.0895-0.0914]	0.4801 [0.4755-0.4844]	0.0029 [0.0012-0.0044]	0.0975 [0.0879-0.1056]
95	Women	0.3468 [0.3424-0.3512]	0.5646 [0.5558-0.5738]	0.0032 [-0.0044-0.0114]	0.0924 [0.0760-0.1091]

Ex: ch stay = excess hazard component due to being a care home resident, Ex: Covid-19 = excess hazard component due to the Covid-19 pandemic experienced by the entire population, Ex: excess = excess excess hazard.
