# Supplementary material for: A new framework for disentangling different components of excess mortality applied to Dutch care home residents during Covid-19
Source: BMC Med Res Methodol. 2025 May 10;25:126. doi: 10.1186/s12874-025-02579-1 (PMC12065346; doi:10.1186/s12874-025-02579-1)
Supplement: Supplementary file 3 — Supplementary Material 3. [file 12874_2025_2579_MOESM3_ESM.pdf]

Supplementary Information: Additional File 3

A new framework for disentangling different components of excess mortality applied to Dutch care home residents during Covid-19

Marije H. Sluiskes[1] (m.h.sluiskes@lumc.nl), Eva A.S. Koster[1], Jelle J. Goeman[1],
Mar Rodríguez-Girondo[1], Hein Putter[1,2], Liesbeth C. de Wreede[1](l.c.de_wreede@lumc.nl)
[1] *Medical Statistics, Biomedical Data Sciences, Leiden University Medical Center, Leiden, The Netherlands*
[2] *Mathematical Institute, Leiden University, Leiden, The Netherlands*

This document contains the R-code used for the main analyses. The code has been copied directly from the R Markdown document.

```
---
title: "Analysis excess excess mortality, age 70 and up"
output:
  html_document:
    toc: true
    toc_float: true
    df_print: paged
date: "Last run: 8 October 2024"
---

In this Rmd-script 5 different models are run, each meant to determine the 4 hazard components:

* Model 1: age effect is categorical
* Model 2: age effect is modeled using splines, with knots at 80 and 90
* Model 3: age effect is modeled using splines, with knot at 85
* Model 4: age effect is modeled using splines, with knots at 75, 85 and 95
* Model 5: age effect is modeled using splines, with knots similar to model 2, but care home variable is excluded from the model

Each section follows a similar structure, with a similar code.

# Set-up

## Load libraries and data

```{r}
library(timereg)
library(survival)
library(tidyverse)
library(mstate)
library(prodlim)
library(ggplot2)
library(stringr)
library(xlsx)
library(splines)
library(gridExtra)
```

```{r}
# small function used to coarsen a dataframe
Coarsen <- function(df, n_timepoints){
  df_coarse <- df[seq(from = 1, to = nrow(df), length.out = n_timepoints),]
```
```

````
  return(df_coarse)
}
```

```{r}
# file 'get_dataset_per_year.Rmd' contains code for preparation of data_allyrs.RData
load("H:/DECIM_Marije/excess_excess/data/data_allyrs.RData")
```

```{r}
# estimate of how many people get VV wlz, per age

# select rows which have (VV) (verpleging en verzorging) in their wlz-description
VV <- grepl("(VV)", data_allyrs$WLZGZZP)
data_allyrs$VV <- VV
# create column which is equal to true if in VV-wlz-verblijf
data_allyrs$in_wlz_verblijf_and_VV <- data_allyrs$in_wlz_verblijf * data_allyrs$VV

t1 <- table(data_allyrs$age_floor)
total <- as.vector(t1)
t2 <- table(data_allyrs$age_floor, data_allyrs$in_wlz_verblijf_and_VV)
in_wlz_verblijf_and_VV <- as.vector(t2[,2])
t3 <- table(data_allyrs$age_floor, data_allyrs$in_wlz_verblijf)
in_wlz_verblijf <- as.vector(t3[,2])

df_overview_wlz_VV <- data.frame(age = names(t1), total, in_wlz_verblijf, in_wlz_verblijf_and_VV)

df_overview_wlz_VV <- df_overview_wlz_VV %>%
  mutate(prop_in_wlz_verblijf_to_total = in_wlz_verblijf / total,
         prop_VV_to_wlz_verblijf = in_wlz_verblijf_and_VV / in_wlz_verblijf)

df_overview_wlz_VV
```

```{r}
# only those aged 70 and up
data_use <- data_allyrs[data_allyrs$age_floor >= 70,]

rm(data_allyrs)
gc()
```

## Numbers reported in the manuscript

```{r}
nrow(data_use)
n_distinct(data_use$RINPERSOON)
sum(data_use$srv_s)
```

## Select subset of data (optional)

```{r}
# Only necessary when not having access to the 'zware desktop' from the CBS
#set.seed(96)
#sample_size <- nrow(data_use)
#data_use_subset <- data_use[sample(1:nrow(data_use), sample_size, replace = F),]

data_use_subset <- data_use
````

rm(data_use)
gc()

data_use_subset$srv=data_use_subset$srv+rnorm(nrow(data_use_subset),0,0.001) # addition of a random small number to break the event time ties
```

# Model 1: age effect categorized

```{r}
data_use_subset$age_cat <- "70-79"
data_use_subset$age_cat[data_use_subset$age_floor >= 80 & data_use_subset$age_floor < 90] <- "80-89"
data_use_subset$age_cat[data_use_subset$age_floor >= 90] <- "90+"
```

M: Fit model

```{r, eval = F}
fitadd_agecat_full_analysis_from70M <- aalen(Surv(entry, srv, srv_s) ~ age_cat +
              in_wlz_verblijf + in_wlz_verblijf:age_cat +
              cov20 + cov20:age_cat +
              cov21 + cov21:age_cat +
              cov20:in_wlz_verblijf + cov20:age_cat:in_wlz_verblijf +
              cov21:in_wlz_verblijf + cov21:age_cat:in_wlz_verblijf,
                data = data_use_subset[data_use_subset$sex == "male",], robust = 0)

save(fitadd_agecat_full_analysis_from70M, file = "../objects/fitadd_agecat_full_analysis_from70M.RData")
gc()
```

W: Fit model

```{r, eval = F}
fitadd_agecat_full_analysis_from70W <- aalen(Surv(entry, srv, srv_s) ~ age_cat +
              in_wlz_verblijf + in_wlz_verblijf:age_cat +
              cov20 + cov20:age_cat +
              cov21 + cov21:age_cat +
              cov20:in_wlz_verblijf + cov20:age_cat:in_wlz_verblijf +
              cov21:in_wlz_verblijf + cov21:age_cat:in_wlz_verblijf,
                data = data_use_subset[data_use_subset$sex == "female",], robust = 0)

save(fitadd_agecat_full_analysis_from70W, file = "../objects/fitadd_agecat_full_analysis_from70W.RData")
gc()
```

Plot results

```{r}
load("H:/DECIM_Marije/excess_excess/additive_hazards/objects/fitadd_agecat_full_analysis_from70M.RData")
load("H:/DECIM_Marije/excess_excess/additive_hazards/objects/fitadd_agecat_full_analysis_from70W.RData")
```

Manually reconstructing the hazards by summing up the relevant model covariates and respective coefficients.

```{r}
## MEN AGED 70-79 ##

```
# time-variable for 2020 and 2021
tplot20 <- fitadd_agecat_full_analysis_from70M$cum[,"time"]
tplot21 <- tplot20 + max(tplot20) - 1 # -1 because Jan 1, 2021 is the 367th day of my model (not the 368th)
tplot <- c(tplot20[-1], tplot21[-1]) # first element not counted because that is 'day 0' when I let everyone enter but no one die
tplot <- tplot[-length(tplot)] # remove last element because 2021 only has 365 days

#   background hazard: intercept + age (NONE: REFERENCE)
bplot20 <- fitadd_agecat_full_analysis_from70M$cum[,"(Intercept)"]
bplot21 <- fitadd_agecat_full_analysis_from70M$cum[,"(Intercept)"] + tail(bplot20, n = 1)
bplot <- c(bplot20[-1], bplot21[-1])
bplot <- bplot[-length(bplot)]

#   wlz-verblijf hazard: wlz-main, wlz:age_cat (NONE: REFERENCE),
wlzplot20 <- fitadd_agecat_full_analysis_from70M$cum[,"in_wlz_verblijf"]
wlzplot21 <- fitadd_agecat_full_analysis_from70M$cum[,"in_wlz_verblijf"] + tail(wlzplot20, n = 1)
wlzplot <- c(wlzplot20[-1], wlzplot21[-1])
wlzplot <- wlzplot[-length(wlzplot)]

#   covid-19 hazard: covid20-main, covid20:age_cat (NONE: REFERENCE) (same for covid21)
cplot20 <- fitadd_agecat_full_analysis_from70M$cum[,"cov20TRUE"]
c21 <- fitadd_agecat_full_analysis_from70M$cum[,"cov21TRUE"]
cplot21 <- c21 + tail(cplot20, n = 1)
cplot <- c(cplot20[-1], cplot21[-1])
cplot <- cplot[-length(cplot)]

#   covid*wlz-verblijf hazard: covid20:wlz, covid20:wlz:age_cat (NONE: REFERENCE) (same for covid21)
eeplot20 <- fitadd_agecat_full_analysis_from70M$cum[,"in_wlz_verblijf:cov20TRUE"]
ee21 <- fitadd_agecat_full_analysis_from70M$cum[,"in_wlz_verblijf:cov21TRUE"]
eeplot21 <- ee21 + tail(eeplot20, n = 1)
eeplot <- c(eeplot20[-1], eeplot21[-1])
eeplot <- eeplot[-length(eeplot)]

df_plotM70_79 <- data.frame(tplot = tplot,
            bplot = bplot,
            wlzplot = wlzplot,
            cplot = cplot,
            eeplot = eeplot,
            age_group = "70-79",
            sex_group = "Men")


## MEN AGED 80-89 ##

# time-variable for 2020 and 2021
tplot20 <- fitadd_agecat_full_analysis_from70M$cum[,1]
tplot21 <- tplot20 + max(tplot20) - 1 # -1 because Jan 1, 2021 is the 367th day of my model (not the 368th)
tplot <- c(tplot20[-1], tplot21[-1]) # first element not counted because that is 'day 0' when I let everyone enter but no one die
tplot <- tplot[-length(tplot)] # remove last element because 2021 only has 365 days

#   background hazard: intercept + age
bplot20 <- fitadd_agecat_full_analysis_from70M$cum[,"(Intercept)"] +
fitadd_agecat_full_analysis_from70M$cum[,"age_cat80-89"]
bplot21 <- fitadd_agecat_full_analysis_from70M$cum[,"(Intercept)"] +
fitadd_agecat_full_analysis_from70M$cum[,"age_cat80-89"] + tail(bplot20, n = 1)
```

```
bplot <- c(bplot20[-1], bplot21[-1])
bplot <- bplot[-length(bplot)]

#   wlz-verblijf hazard: wlz-main, wlz:age_cat
wlzplot20 <- fitadd_agecat_full_analysis_from70M$cum[,"in_wlz_verblijf"] +
fitadd_agecat_full_analysis_from70M$cum[,"age_cat80-89:in_wlz_verblijf"]
wlzplot21 <- fitadd_agecat_full_analysis_from70M$cum[,"in_wlz_verblijf"] +
fitadd_agecat_full_analysis_from70M$cum[,"age_cat80-89:in_wlz_verblijf"] + tail(wlzplot20, n = 1)
wlzplot <- c(wlzplot20[-1], wlzplot21[-1])
wlzplot <- wlzplot[-length(wlzplot)]

#   covid-19 hazard: covid20-main, covid20:age_cat (same for covid21)
cplot20 <- fitadd_agecat_full_analysis_from70M$cum[,"cov20TRUE"]  +
fitadd_agecat_full_analysis_from70M$cum[,"age_cat80-89:cov20TRUE"]
c21 <- fitadd_agecat_full_analysis_from70M$cum[,"cov21TRUE"]  +
fitadd_agecat_full_analysis_from70M$cum[,"age_cat80-89:cov21TRUE"]
cplot21 <- c21 + tail(cplot20, n = 1)
cplot <- c(cplot20[-1], cplot21[-1])
cplot <- cplot[-length(cplot)]

#   covid*wlz-verblijf hazard: covid20:wlz, covid20:wlz:age_cat (same for covid21)
eeplot20 <- fitadd_agecat_full_analysis_from70M$cum[,"in_wlz_verblijf:cov20TRUE"] +
fitadd_agecat_full_analysis_from70M$cum[,"age_cat80-89:in_wlz_verblijf:cov20TRUE"]
ee21 <- fitadd_agecat_full_analysis_from70M$cum[,"in_wlz_verblijf:cov21TRUE"] +
fitadd_agecat_full_analysis_from70M$cum[,"age_cat80-89:in_wlz_verblijf:cov21TRUE"]
eeplot21 <- ee21 + tail(eeplot20, n = 1)
eeplot <- c(eeplot20[-1], eeplot21[-1])
eeplot <- eeplot[-length(eeplot)]

df_plotM80_89 <- data.frame(tplot = tplot,
                bplot = bplot,
                wlzplot = wlzplot,
                cplot = cplot,
                eeplot = eeplot,
                age_group = "80-89",
                sex_group = "Men")

## MEN AGED 90+ ##

# time-variable for 2020 and 2021
tplot20 <- fitadd_agecat_full_analysis_from70M$cum[,1]
tplot21 <- tplot20 + max(tplot20) - 1 # -1 because Jan 1, 2021 is the 367th day of my model (not the
368th)
tplot <- c(tplot20[-1], tplot21[-1]) # first element not counted because that is 'day 0' when I let everyone
enter but no one die
tplot <- tplot[-length(tplot)] # remove last element because 2021 only has 365 days

#   background hazard: intercept + age
bplot20 <- fitadd_agecat_full_analysis_from70M$cum[,"(Intercept)"] +
fitadd_agecat_full_analysis_from70M$cum[,"age_cat90+"]
bplot21 <- fitadd_agecat_full_analysis_from70M$cum[,"(Intercept)"] +
fitadd_agecat_full_analysis_from70M$cum[,"age_cat90+"] + tail(bplot20, n = 1)
bplot <- c(bplot20[-1], bplot21[-1])
bplot <- bplot[-length(bplot)]

#   wlz-verblijf hazard: wlz-main, wlz:age_cat
wlzplot20 <- fitadd_agecat_full_analysis_from70M$cum[,"in_wlz_verblijf"] +
fitadd_agecat_full_analysis_from70M$cum[,"age_cat90+:in_wlz_verblijf"]
```

~~~
wlzplot21 <- fitadd_agecat_full_analysis_from70M$cum[,"in_wlz_verblijf"] +
fitadd_agecat_full_analysis_from70M$cum[,"age_cat90+:in_wlz_verblijf"]+ tail(wlzplot20, n = 1)
wlzplot <- c(wlzplot20[-1], wlzplot21[-1])
wlzplot <- wlzplot[-length(wlzplot)]

#   covid-19 hazard: covid20-main, covid20:age_cat (same for covid21)
cplot20 <- fitadd_agecat_full_analysis_from70M$cum[,"cov20TRUE"]  +
fitadd_agecat_full_analysis_from70M$cum[,"age_cat90+:cov20TRUE"]
c21 <- fitadd_agecat_full_analysis_from70M$cum[,"cov21TRUE"]  +
fitadd_agecat_full_analysis_from70M$cum[,"age_cat90+:cov21TRUE"]
cplot21 <- c21 + tail(cplot20, n = 1)
cplot <- c(cplot20[-1], cplot21[-1])
cplot <- cplot[-length(cplot)]

#   covid*wlz-verblijf hazard: covid20:wlz, covid20:wlz:age_cat (same for covid21)
eeplot20 <- fitadd_agecat_full_analysis_from70M$cum[,"in_wlz_verblijf:cov20TRUE"] +
fitadd_agecat_full_analysis_from70M$cum[,"age_cat90+:in_wlz_verblijf:cov20TRUE"]
ee21 <- fitadd_agecat_full_analysis_from70M$cum[,"in_wlz_verblijf:cov21TRUE"] +
fitadd_agecat_full_analysis_from70M$cum[,"age_cat90+:in_wlz_verblijf:cov21TRUE"]
eeplot21 <- ee21 + tail(eeplot20, n = 1)
eeplot <- c(eeplot20[-1], eeplot21[-1])
eeplot <- eeplot[-length(eeplot)]

df_plotM90plus <- data.frame(tplot = tplot,
                bplot = bplot,
                wlzplot = wlzplot,
                cplot = cplot,
                eeplot = eeplot,
                age_group = "90+",
                sex_group = "Men")


```


```{r}
## WOMEN AGED 70-79 ##

# time-variable for 2020 and 2021
tplot20 <- fitadd_agecat_full_analysis_from70W$cum[,"time"]
tplot21 <- tplot20 + max(tplot20) - 1 # -1 because Jan 1, 2021 is the 367th day of my model (not the
368th)
tplot <- c(tplot20[-1], tplot21[-1]) # first element not counted because that is 'day 0' when I let everyone
enter but no one die
tplot <- tplot[-length(tplot)] # remove last element because 2021 only has 365 days

#   background hazard: intercept + age (NONE: REFERENCE)
bplot20 <- fitadd_agecat_full_analysis_from70W$cum[,"(Intercept)"]
bplot21 <- fitadd_agecat_full_analysis_from70W$cum[,"(Intercept)"] + tail(bplot20, n = 1)
bplot <- c(bplot20[-1], bplot21[-1])
bplot <- bplot[-length(bplot)]

#   wlz-verblijf hazard: wlz-main, wlz:age_cat (NONE: REFERENCE),
wlzplot20 <- fitadd_agecat_full_analysis_from70W$cum[,"in_wlz_verblijf"]
wlzplot21 <- fitadd_agecat_full_analysis_from70W$cum[,"in_wlz_verblijf"] + tail(wlzplot20, n = 1)
wlzplot <- c(wlzplot20[-1], wlzplot21[-1])
wlzplot <- wlzplot[-length(wlzplot)]

#   covid-19 hazard: covid20-main, covid20:age_cat (NONE: REFERENCE) (same for covid21)
cplot20 <- fitadd_agecat_full_analysis_from70W$cum[,"cov20TRUE"]
~~~

```
c21 <- fitadd_agecat_full_analysis_from70W$cum[,"cov21TRUE"]
cplot21 <- c21 + tail(cplot20, n = 1)
cplot <- c(cplot20[-1], cplot21[-1])
cplot <- cplot[-length(cplot)]

#   covid*wlz-verblijf hazard: covid20:wlz, covid20:wlz:age_cat (NONE: REFERENCE) (same for covid21)
eeplot20 <- fitadd_agecat_full_analysis_from70W$cum[,"in_wlz_verblijf:cov20TRUE"]
ee21 <- fitadd_agecat_full_analysis_from70W$cum[,"in_wlz_verblijf:cov21TRUE"]
eeplot21 <- ee21 + tail(eeplot20, n = 1)
eeplot <- c(eeplot20[-1], eeplot21[-1])
eeplot <- eeplot[-length(eeplot)]

df_plotW70_79 <- data.frame(tplot = tplot,
                 bplot = bplot,
                 wlzplot = wlzplot,
                 cplot = cplot,
                 eeplot = eeplot,
                 age_group = "70-79",
                 sex_group = "Women")


## WOMEN AGED 80-89 ##

# time-variable for 2020 and 2021
tplot20 <- fitadd_agecat_full_analysis_from70W$cum[,1]
tplot21 <- tplot20 + max(tplot20) - 1 # -1 because Jan 1, 2021 is the 367th day of my model (not the 368th)
tplot <- c(tplot20[-1], tplot21[-1]) # first element not counted because that is 'day 0' when I let everyone enter but no one die
tplot <- tplot[-length(tplot)] # remove last element because 2021 only has 365 days

#   background hazard: intercept + age
bplot20 <- fitadd_agecat_full_analysis_from70W$cum[,"(Intercept)"] + fitadd_agecat_full_analysis_from70W$cum[,"age_cat80-89"]
bplot21 <- fitadd_agecat_full_analysis_from70W$cum[,"(Intercept)"] + fitadd_agecat_full_analysis_from70W$cum[,"age_cat80-89"] + tail(bplot20, n = 1)
bplot <- c(bplot20[-1], bplot21[-1])
bplot <- bplot[-length(bplot)]

#   wlz-verblijf hazard: wlz-main, wlz:age_cat
wlzplot20 <- fitadd_agecat_full_analysis_from70W$cum[,"in_wlz_verblijf"] + fitadd_agecat_full_analysis_from70W$cum[,"age_cat80-89:in_wlz_verblijf"]
wlzplot21 <- fitadd_agecat_full_analysis_from70W$cum[,"in_wlz_verblijf"] + fitadd_agecat_full_analysis_from70W$cum[,"age_cat80-89:in_wlz_verblijf"] + tail(wlzplot20, n = 1)
wlzplot <- c(wlzplot20[-1], wlzplot21[-1])
wlzplot <- wlzplot[-length(wlzplot)]

#   covid-19 hazard: covid20-main, covid20:age_cat (same for covid21)
cplot20 <- fitadd_agecat_full_analysis_from70W$cum[,"cov20TRUE"]  + fitadd_agecat_full_analysis_from70W$cum[,"age_cat80-89:cov20TRUE"]
c21 <- fitadd_agecat_full_analysis_from70W$cum[,"cov21TRUE"]  + fitadd_agecat_full_analysis_from70W$cum[,"age_cat80-89:cov21TRUE"]
cplot21 <- c21 + tail(cplot20, n = 1)
cplot <- c(cplot20[-1], cplot21[-1])
cplot <- cplot[-length(cplot)]

#   covid*wlz-verblijf hazard: covid20:wlz, covid20:wlz:age_cat (same for covid21)
eeplot20 <- fitadd_agecat_full_analysis_from70W$cum[,"in_wlz_verblijf:cov20TRUE"] + fitadd_agecat_full_analysis_from70W$cum[,"age_cat80-89:in_wlz_verblijf:cov20TRUE"]
```

```
ee21 <- fitadd_agecat_full_analysis_from70W$cum[,"in_wlz_verblijf:cov21TRUE"] +
fitadd_agecat_full_analysis_from70W$cum[,"age_cat80-89:in_wlz_verblijf:cov21TRUE"]
eeplot21 <- ee21 + tail(eeplot20, n = 1)
eeplot <- c(eeplot20[-1], eeplot21[-1])
eeplot <- eeplot[-length(eeplot)]

df_plotW80_89 <- data.frame(tplot = tplot,
                bplot = bplot,
                wlzplot = wlzplot,
                cplot = cplot,
                eeplot = eeplot,
                age_group = "80-89",
                sex_group = "Women")

## WOMEN AGED 90+ ##

# time-variable for 2020 and 2021
tplot20 <- fitadd_agecat_full_analysis_from70W$cum[,1]
tplot21 <- tplot20 + max(tplot20) - 1 # -1 because Jan 1, 2021 is the 367th day of my model (not the
368th)
tplot <- c(tplot20[-1], tplot21[-1]) # first element not counted because that is 'day 0' when I let everyone
enter but no one die
tplot <- tplot[-length(tplot)] # remove last element because 2021 only has 365 days

#   background hazard: intercept + age
bplot20 <- fitadd_agecat_full_analysis_from70W$cum[,"(Intercept)"] +
fitadd_agecat_full_analysis_from70W$cum[,"age_cat90+"]
bplot21 <- fitadd_agecat_full_analysis_from70W$cum[,"(Intercept)"] +
fitadd_agecat_full_analysis_from70W$cum[,"age_cat90+"] + tail(bplot20, n = 1)
bplot <- c(bplot20[-1], bplot21[-1])
bplot <- bplot[-length(bplot)]

#   wlz-verblijf hazard: wlz-main, wlz:age_cat
wlzplot20 <- fitadd_agecat_full_analysis_from70W$cum[,"in_wlz_verblijf"] +
fitadd_agecat_full_analysis_from70W$cum[,"age_cat90+:in_wlz_verblijf"]
wlzplot21 <- fitadd_agecat_full_analysis_from70W$cum[,"in_wlz_verblijf"] +
fitadd_agecat_full_analysis_from70W$cum[,"age_cat90+:in_wlz_verblijf"]+ tail(wlzplot20, n = 1)
wlzplot <- c(wlzplot20[-1], wlzplot21[-1])
wlzplot <- wlzplot[-length(wlzplot)]

#   covid-19 hazard: covid20-main, covid20:age_cat (same for covid21)
cplot20 <- fitadd_agecat_full_analysis_from70W$cum[,"cov20TRUE"]  +
fitadd_agecat_full_analysis_from70W$cum[,"age_cat90+:cov20TRUE"]
c21 <- fitadd_agecat_full_analysis_from70W$cum[,"cov21TRUE"]  +
fitadd_agecat_full_analysis_from70W$cum[,"age_cat90+:cov21TRUE"]
cplot21 <- c21 + tail(cplot20, n = 1)
cplot <- c(cplot20[-1], cplot21[-1])
cplot <- cplot[-length(cplot)]

#   covid*wlz-verblijf hazard: covid20:wlz, covid20:wlz:age_cat (same for covid21)
eeplot20 <- fitadd_agecat_full_analysis_from70W$cum[,"in_wlz_verblijf:cov20TRUE"] +
fitadd_agecat_full_analysis_from70W$cum[,"age_cat90+:in_wlz_verblijf:cov20TRUE"]
ee21 <- fitadd_agecat_full_analysis_from70W$cum[,"in_wlz_verblijf:cov21TRUE"] +
fitadd_agecat_full_analysis_from70W$cum[,"age_cat90+:in_wlz_verblijf:cov21TRUE"]
eeplot21 <- ee21 + tail(eeplot20, n = 1)
eeplot <- c(eeplot20[-1], eeplot21[-1])
eeplot <- eeplot[-length(eeplot)]

df_plotW90plus <- data.frame(tplot = tplot,
```

````
                 bplot = bplot,
                 wlzplot = wlzplot,
                 cplot = cplot,
                 eeplot = eeplot,
                 age_group = "90+",
                 sex_group = "Women")


```

```{r}
# coarsening, to have fewer datapoints. speeds up plotting and makes exporting easier.
n_timepoints <- 2000
df_plotW70_79_coarse <- Coarsen(df_plotW70_79, n_timepoints)
df_plotM70_79_coarse <- Coarsen(df_plotM70_79, n_timepoints)
df_plotW80_89_coarse <- Coarsen(df_plotW80_89, n_timepoints)
df_plotM80_89_coarse <- Coarsen(df_plotM80_89, n_timepoints)
df_plotW90plus_coarse <- Coarsen(df_plotW90plus, n_timepoints)
df_plotM90plus_coarse <- Coarsen(df_plotM90plus, n_timepoints)

df_plothaz_agecat <- rbind(df_plotW70_79_coarse, df_plotM70_79_coarse,
             df_plotW80_89_coarse, df_plotM80_89_coarse,
             df_plotW90plus_coarse, df_plotM90plus_coarse)

p.allhaz.grid.agecat <- df_plothaz_agecat %>%
  ggplot(aes(x = tplot)) +
  geom_line(aes(y = bplot, col = "blue")) +
  geom_line(aes(y = wlzplot, col = "orange")) +
  geom_line(aes(y = cplot, col = "purple")) +
  geom_line(aes(y = eeplot, col = "red")) +
  labs(x = "Days (2020 + 2021)", y = "Cumulative hazard", title = "All age-effects categorical") +
  scale_color_manual(name = "Mortality components", values = c("orange" = "orange", "blue" = "blue",
"purple" = "purple", "red" = "red"), labels = c("Background", "Excess: care home stay", "Excess: Covid-19
pandemic", "Excess: excess")) +
  facet_grid(sex_group~age_group) +
  coord_cartesian(ylim = c(0, 0.9))
p.allhaz.grid.agecat
ggsave(p.allhaz.grid.agecat, file = "../figures/p.allhaz.grid.agecat.full_analysis_from70.png",
width=10,height=4,dpi=300)

p.c.grid.agecat <-df_plothaz_agecat %>%
  ggplot(aes(x = tplot)) +
  geom_line(aes(y = cplot, col = "purple")) +
  labs(x = "Days (2020 + 2021)", y = "Cumulative hazard", title = "All age-effects categorical") +
  scale_color_manual(name = "Mortality components", values = c("purple" = "purple"), labels = c("Excess:
Covid-19 pandemic")) +
  facet_grid(sex_group~age_group) +
  coord_cartesian(ylim = c(-0.005, 0.03))
p.c.grid.agecat

```
````

Plot age effect

To check if the effect of age is linear, and if not, which shape.

````
```{r}
GetCatAgeEffect <- function(sexvar, agevec, timepoint, fitted_model){

  b_cumhaz_age <- case_when(agevec < 80 ~0,
````

```
                      agevec >=80 & agevec < 90 ~ fitted_model$cum[timepoint ,"age_cat80-89"],
                      .default = fitted_model$cum[timepoint ,"age_cat90+"]
                      )

  wlz_cumhaz_age <- case_when(agevec < 80 ~0,
                      agevec >=80 & agevec < 90 ~ fitted_model$cum[timepoint,"age_cat80-
89:in_wlz_verblijf"],
                      .default = fitted_model$cum[timepoint,"age_cat90+:in_wlz_verblijf"]
                      )

  c20_cumhaz_age <- case_when(agevec < 80 ~0,
                      agevec >=80 & agevec < 90 ~ fitted_model$cum[timepoint,"age_cat80-89:cov20TRUE"],
                      .default = fitted_model$cum[timepoint,"age_cat90+:cov20TRUE"]
                      )

  c21_cumhaz_age <- case_when(agevec < 80 ~0,
                      agevec >=80 & agevec < 90 ~ fitted_model$cum[timepoint,"age_cat80-89:cov21TRUE"],
                      .default = fitted_model$cum[timepoint,"age_cat90+:cov21TRUE"]
                      )

  ee20_cumhaz_age <- case_when(agevec < 80 ~0,
                      agevec >=80 & agevec < 90 ~ fitted_model$cum[timepoint,"age_cat80-
89:in_wlz_verblijf:cov20TRUE"],
                      .default = fitted_model$cum[timepoint,"age_cat90+:in_wlz_verblijf:cov20TRUE"]
                      )

  ee21_cumhaz_age <- case_when(agevec < 80 ~0,
                      agevec >=80 & agevec < 90 ~ fitted_model$cum[timepoint,"age_cat80-
89:in_wlz_verblijf:cov21TRUE"],
                      .default = fitted_model$cum[timepoint,"age_cat90+:in_wlz_verblijf:cov21TRUE"]
                      )

  df_plot <- data.frame(cbind(age = agevec, b_cumhaz_age, wlz_cumhaz_age, c20_cumhaz_age,
c21_cumhaz_age, ee20_cumhaz_age, ee21_cumhaz_age))
  return(df_plot)

}
```

Plotting effect of age at 3 different timepoints (day 100, 200 and 300).

```{r}
my_age <- 70:100

## TIMEPOINT 1 ##
# time-variable for 2020 and 2021
my_time <- 100
my_timepoint <- which.min(abs(fitadd_agecat_full_analysis_from70M$cum[,1] - my_time))

df_plotW <- GetCatAgeEffect(sexvar = 0, agevec = my_age, timepoint = my_timepoint, fitted_model =
fitadd_agecat_full_analysis_from70W)
df_plotW$sex <- "Women"
df_plotM <- GetCatAgeEffect(sexvar = 1, agevec = my_age, timepoint = my_timepoint, fitted_model =
fitadd_agecat_full_analysis_from70M)
df_plotM$sex <- "Men"

df_plotage_agecat_t100  <- rbind(df_plotW, df_plotM)

p.agecat.age.effect.t100 <- df_plotage_agecat_t100  %>%
  ggplot(aes(x = age)) +
```

```
  geom_step(aes(y = b_cumhaz_age, col = "blue")) +
  geom_step(aes(y = wlz_cumhaz_age, col = "orange")) +
  geom_step(aes(y = c20_cumhaz_age, col = "purple")) +
  geom_step(aes(y = c21_cumhaz_age, col = "purple4")) +
  geom_step(aes(y = ee20_cumhaz_age, col = "red")) +
  geom_step(aes(y = ee21_cumhaz_age, col = "red4")) +
  facet_grid(~sex) +
  labs(x = "Age", y = "Cumulative hazard", title = "Age effect (categorical), time = day 100") +
  scale_color_manual(name = "Mortality components",
                values = c("orange" = "orange", "blue" = "blue", "purple" = "purple", "purple4" = "purple4",
"red" = "red", "red4" = "red4"),
                labels = c("blue" = "Background", "orange" = "Excess: care home stay",
                        "purple"= "Excess: Covid-19 pandemic (2020)","purple4"="Excess: Covid-19 pandemic
(2021)",
                        "red"= "Excess: excess (2020)", "red4"= "Excess: excess (2021)"))
p.agecat.age.effect.t100
ggsave(p.agecat.age.effect.t100, file = "../figures/p.agecat.age.effect.t100.full_analysis_from70.png",
width=10,height=4,dpi=300)

## TIMEPOINT 2 ##
# time-variable for 2020 and 2021
my_time <- 200
my_timepoint <- which.min(abs(fitadd_agecat_full_analysis_from70M$cum[,1] - my_time))

df_plotW <- GetCatAgeEffect(sexvar = 0, agevec = my_age, timepoint = my_timepoint, fitted_model =
fitadd_agecat_full_analysis_from70W)
df_plotW$sex <- "Women"
df_plotM <- GetCatAgeEffect(sexvar = 1, agevec = my_age, timepoint = my_timepoint, fitted_model =
fitadd_agecat_full_analysis_from70M)
df_plotM$sex <- "Men"

df_plotage_agecat_t200  <- rbind(df_plotW, df_plotM)

p.agecat.age.effect.t200 <- df_plotage_agecat_t200  %>%
  ggplot(aes(x = age)) +
  geom_step(aes(y = b_cumhaz_age, col = "blue")) +
  geom_step(aes(y = wlz_cumhaz_age, col = "orange")) +
  geom_step(aes(y = c20_cumhaz_age, col = "purple")) +
  geom_step(aes(y = c21_cumhaz_age, col = "purple4")) +
  geom_step(aes(y = ee20_cumhaz_age, col = "red")) +
  geom_step(aes(y = ee21_cumhaz_age, col = "red4")) +
  facet_grid(~sex) +
  labs(x = "Age", y = "Cumulative hazard", title = "Age effect (categorical), time = day 200") +
  scale_color_manual(name = "Mortality components",
                values = c("orange" = "orange", "blue" = "blue", "purple" = "purple", "purple4" = "purple4",
"red" = "red", "red4" = "red4"),
                labels = c("blue" = "Background", "orange" = "Excess: care home stay",
                        "purple"= "Excess: Covid-19 pandemic (2020)","purple4"="Excess: Covid-19 pandemic
(2021)",
                        "red"= "Excess: excess (2020)", "red4"= "Excess: excess (2021)"))
p.agecat.age.effect.t200
ggsave(p.agecat.age.effect.t200, file = "../figures/p.agecat.age.effect.t200.full_analysis_from70.png",
width=10,height=4,dpi=300)

## TIMEPOINT 3 ##
# time-variable for 2020 and 2021
my_time <- 300
my_timepoint <- which.min(abs(fitadd_agecat_full_analysis_from70M$cum[,1] - my_time))
```

~~~
df_plotW <- GetCatAgeEffect(sexvar = 0, agevec = my_age, timepoint = my_timepoint, fitted_model = fitadd_agecat_full_analysis_from70W)
df_plotW$sex <- "Women"
df_plotM <- GetCatAgeEffect(sexvar = 1, agevec = my_age, timepoint = my_timepoint, fitted_model = fitadd_agecat_full_analysis_from70M)
df_plotM$sex <- "Men"

df_plotage_agecat_t300 <- rbind(df_plotW, df_plotM)

p.agecat.age.effect.t300 <- df_plotage_agecat_t300  %>%
  ggplot(aes(x = age)) +
  geom_step(aes(y = b_cumhaz_age, col = "blue")) +
  geom_step(aes(y = wlz_cumhaz_age, col = "orange")) +
  geom_step(aes(y = c20_cumhaz_age, col = "purple")) +
  geom_step(aes(y = c21_cumhaz_age, col = "purple4")) +
  geom_step(aes(y = ee20_cumhaz_age, col = "red")) +
  geom_step(aes(y = ee21_cumhaz_age, col = "red4")) +
  facet_grid(~sex) +
  labs(x = "Age", y = "Cumulative hazard", title = "Age effect (categorical), time = day 300") +
  scale_color_manual(name = "Mortality components",
                     values = c("orange" = "orange", "blue" = "blue", "purple" = "purple", "purple4" = "purple4", "red" = "red", "red4" = "red4"),
                     labels = c("blue" = "Background", "orange" = "Excess: care home stay",
                                "purple"= "Excess: Covid-19 pandemic (2020)","purple4"="Excess: Covid-19 pandemic (2021)",
                                "red"= "Excess: excess (2020)", "red4"= "Excess: excess (2021)"))
p.agecat.age.effect.t300
ggsave(p.agecat.age.effect.t300, file = "../figures/p.agecat.age.effect.t300.full_analysis_from70.png", width=10,height=4,dpi=300)

```
~~~

Model 2: age effect splines (knots 80, 90)

We start by fitting a natural cubic spline with 2 knots (and 2 boundary knots), located at the cut-off points of the categorized age we considered in the analysis above.

~~~
```{r}
my_knots <- c(80,90)
my_boundary_knots <- c(70, 100) # necessary

spline_age <- ns(data_use_subset$age_floor, knots = my_knots, Boundary.knots = my_boundary_knots)
```
~~~

M: Fit model

~~~
```{r, eval = F}
spline_age <- ns(data_use_subset$age_floor, knots = my_knots, Boundary.knots = my_boundary_knots)
spline_age <- spline_age[data_use_subset$sex == "male",] # temporary, to get correct covariate names in model
gc()

fitadd_agespline_full_analysis_from70M <- aalen(Surv(entry, srv, srv_s) ~
                        spline_age +
              in_wlz_verblijf + in_wlz_verblijf:spline_age +
              cov20 + cov20:spline_age +
              cov21 + cov21:spline_age +
              cov20:in_wlz_verblijf + cov20:spline_age:in_wlz_verblijf +
              cov21:in_wlz_verblijf + cov21:spline_age:in_wlz_verblijf,
                data = data_use_subset[data_use_subset$sex == "male",], robust = 0)
~~~

save(fitadd_agespline_full_analysis_from70M, file = "../objects/fitadd_agespline_full_analysis_from70M.RData")
spline_age <- ns(data_use_subset$age_floor, knots = my_knots, Boundary.knots = my_boundary_knots)
```

## W: Fit model
```{r, eval = F}
spline_age <- ns(data_use_subset$age_floor, knots = my_knots, Boundary.knots = my_boundary_knots)
spline_age <- spline_age[data_use_subset$sex == "female",] # temporary, to get correct covariate names in model
gc()

fitadd_agespline_full_analysis_from70W <- aalen(Surv(entry, srv, srv_s) ~
                        spline_age +
              in_wlz_verblijf + in_wlz_verblijf:spline_age +
              cov20 + cov20:spline_age +
              cov21 + cov21:spline_age +
              cov20:in_wlz_verblijf + cov20:spline_age:in_wlz_verblijf +
              cov21:in_wlz_verblijf + cov21:spline_age:in_wlz_verblijf,
                data = data_use_subset[data_use_subset$sex == "female",], robust = 0)


save(fitadd_agespline_full_analysis_from70W, file = "../objects/fitadd_agespline_full_analysis_from70W.RData")
spline_age <- ns(data_use_subset$age_floor, knots = my_knots, Boundary.knots = my_boundary_knots)
```

Plot results

```{r}
gc()

load("H:/DECIM_Marije/excess_excess/additive_hazards/objects/fitadd_agespline_full_analysis_from70M.RData")
load("H:/DECIM_Marije/excess_excess/additive_hazards/objects/fitadd_agespline_full_analysis_from70W.RData")

spline_age <- ns(data_use_subset$age_floor, knots = my_knots, Boundary.knots = my_boundary_knots)
```

```{r}
GetHazards <- function(agevar, fitted_model, splinepred){

   # time-variable for 2020 and 2021
   tplot20 <- fitted_model$cum[,1]
   tplot21 <- tplot20 + max(tplot20) - 1 # -1 because Jan 1, 2021 is the 367th day of my model (not the 368th)
   tplot <- c(tplot20[-1], tplot21[-1]) # first element not counted because that is 'day 0' when I let everyone enter but no one die
   tplot <- tplot[-length(tplot)] # remove last element because 2021 only has 365 days

   #   background hazard: intercept + age
   bplot20 <- fitted_model$cum[,"(Intercept)"] +
     fitted_model$cum[,"spline_age1"] * splinepred[1] +
     fitted_model$cum[,"spline_age2"] * splinepred[2] +
     fitted_model$cum[,"spline_age3"] * splinepred[3]

```
  bplot21 <- bplot20 + tail(bplot20, 1)
  bplot <- c(bplot20[-1], bplot21[-1])
  bplot <- bplot[-length(bplot)]

  #   wlz-verblijf hazard: wlz-main, wlz:age
  wlzplot20 <- fitted_model$cum[,"in_wlz_verblijf"] +
    fitted_model$cum[,"spline_age1:in_wlz_verblijf"] * splinepred[1] +
    fitted_model$cum[,"spline_age2:in_wlz_verblijf"] * splinepred[2] +
    fitted_model$cum[,"spline_age3:in_wlz_verblijf"] * splinepred[3]

  wlzplot21 <- wlzplot20 + tail(wlzplot20, 1)
  wlzplot <- c(wlzplot20[-1], wlzplot21[-1])
  wlzplot <- wlzplot[-length(wlzplot)]

  #   covid-19 hazard: covid20-main, covid20:age_cat (same for covid21)
  cplot20 <- fitted_model$cum[,"cov20TRUE"] +
    fitted_model$cum[,"spline_age1:cov20TRUE"] * splinepred[1] +
    fitted_model$cum[,"spline_age2:cov20TRUE"] * splinepred[2] +
    fitted_model$cum[,"spline_age3:cov20TRUE"] * splinepred[3]

  c21 <- fitted_model$cum[,"cov21TRUE"] +
    fitted_model$cum[,"spline_age1:cov21TRUE"] * splinepred[1] +
    fitted_model$cum[,"spline_age2:cov21TRUE"] * splinepred[2] +
    fitted_model$cum[,"spline_age3:cov21TRUE"] * splinepred[3]

  cplot21 <- c21 + tail(cplot20, n = 1)
  cplot <- c(cplot20[-1], cplot21[-1])
  cplot <- cplot[-length(cplot)]

  #   covid*wlz-verblijf hazard: covid20:wlz, covid20:wlz:age (same for covid21)
  eeplot20 <- fitted_model$cum[,"in_wlz_verblijf:cov20TRUE"] +
    fitted_model$cum[,"spline_age1:in_wlz_verblijf:cov20TRUE"] * splinepred[1] +
    fitted_model$cum[,"spline_age2:in_wlz_verblijf:cov20TRUE"] * splinepred[2] +
    fitted_model$cum[,"spline_age3:in_wlz_verblijf:cov20TRUE"] * splinepred[3]

  ee21 <- fitted_model$cum[,"in_wlz_verblijf:cov21TRUE"] +
    fitted_model$cum[,"spline_age1:in_wlz_verblijf:cov21TRUE"] * splinepred[1] +
    fitted_model$cum[,"spline_age2:in_wlz_verblijf:cov21TRUE"] * splinepred[2] +
    fitted_model$cum[,"spline_age3:in_wlz_verblijf:cov21TRUE"] * splinepred[3]

  eeplot21 <- ee21 + tail(eeplot20, n = 1)
  eeplot <- c(eeplot20[-1], eeplot21[-1])
  eeplot <- eeplot[-length(eeplot)]

  df_plot <- data.frame(tplot = tplot,
                        bplot = bplot,
                        wlzplot = wlzplot,
                        cplot = cplot,
                        eeplot = eeplot,
                        age = agevar)

  return(df_plot)
}
```

```{r}
my_agevars = c(75, 85, 95)

## Men ##
```

```
df_plothaz_agespline2_listM <- list()

for (i in 1:length(my_agevars)){

  my_splinepred <- predict(spline_age, my_agevars[i])[1,]
  df_plothaz_agespline2 <- GetHazards(agevar = my_agevars[i], fitted_model =
fitadd_agespline_full_analysis_from70M, splinepred = my_splinepred)
  df_plothaz_agespline2_listM[[i]] <- df_plothaz_agespline2
}

df_plothaz_agespline2_list_coarsenedM <- lapply(df_plothaz_agespline2_listM, Coarsen, n_timepoints =
2000)
df_plothaz_agespline2M <- list_rbind(df_plothaz_agespline2_list_coarsenedM)

df_plothaz_agespline2M$sex <- "Men"

## Women ##

df_plothaz_agespline2_listW <- list()

for (i in 1:length(my_agevars)){

  my_splinepred <- predict(spline_age, my_agevars[i])[1,]
  df_plothaz_agespline2 <- GetHazards(agevar = my_agevars[i], fitted_model =
fitadd_agespline_full_analysis_from70W, splinepred = my_splinepred)
  df_plothaz_agespline2_listW[[i]] <- df_plothaz_agespline2
}

df_plothaz_agespline2_list_coarsenedW <- lapply(df_plothaz_agespline2_listW, Coarsen, n_timepoints =
2000)
df_plothaz_agespline2W <- list_rbind(df_plothaz_agespline2_list_coarsenedW)

df_plothaz_agespline2W$sex <- "Women"

df_plothaz_agespline2 <- rbind(df_plothaz_agespline2M, df_plothaz_agespline2W)


p.allhaz.grid.agespline <- df_plothaz_agespline2 %>%
  ggplot(aes(x = tplot)) +
  geom_line(aes(y = bplot, col = "blue")) +
  geom_line(aes(y = wlzplot, col = "orange")) +
  geom_line(aes(y = cplot, col = "purple")) +
  geom_line(aes(y = eeplot, col = "red")) +
  labs(x = "Days (2020 + 2021)", y = "Cumulative hazard", title = "Age with splines (knots at 80, 90)") +
  scale_color_manual(name = "Mortality components", values = c("orange" = "orange", "blue" = "blue",
"purple" = "purple", "red" = "red"), labels = c("Background", "Excess: care home stay", "Excess: Covid-19
pandemic", "Excess: excess")) +
  facet_grid(sex~age) +
  coord_cartesian(ylim = c(-0.1, 0.9))
p.allhaz.grid.agespline

ggsave(p.allhaz.grid.agespline, file = "../figures/p.allhaz.grid.agespline.full_analysis_from70.png",
width=10,height=4,dpi=300)

```

Plot age effect

Function to obtain the effect of age at a given timepoint.

````
```{r}
GetAgeEffect <- function(agevec, timepoint, fitted_model, spline_age){

 b_cumhaz_age <- vector(length = length(agevec))
 wlz_cumhaz_age <- vector(length = length(agevec))
 c20_cumhaz_age <- vector(length = length(agevec))
 c21_cumhaz_age <- vector(length = length(agevec))
 ee20_cumhaz_age <- vector(length = length(agevec))
 ee21_cumhaz_age <- vector(length = length(agevec))

 for (i in 1:length(agevec)){

   splinepred <- predict(spline_age, agevec[i])[1,]

   # background hazard age-effect
   bplot_cumhaz_age <- fitted_model$cum[timepoint,"spline_age1"] * splinepred[1] +
     fitted_model$cum[timepoint,"spline_age2"] * splinepred[2] +
     fitted_model$cum[timepoint,"spline_age3"] * splinepred[3]

    b_cumhaz_age[i] <- bplot_cumhaz_age

   # wlz-verblijf hazard age-effect
     wlzplot_cumhaz_age <- fitted_model$cum[timepoint,"spline_age1:in_wlz_verblijf"] * splinepred[1] +
       fitted_model$cum[timepoint,"spline_age2:in_wlz_verblijf"] * splinepred[2] +
       fitted_model$cum[timepoint,"spline_age3:in_wlz_verblijf"] * splinepred[3]

     wlz_cumhaz_age[i] <- wlzplot_cumhaz_age

     # covid-19 hazard age-effect
     cplot20_cumhaz_age <- fitted_model$cum[timepoint,"spline_age1:cov20TRUE"] * splinepred[1] +
       fitted_model$cum[timepoint,"spline_age2:cov20TRUE"] * splinepred[2] +
       fitted_model$cum[timepoint,"spline_age3:cov20TRUE"] * splinepred[3]

     cplot21_cumhaz_age <- fitted_model$cum[timepoint,"spline_age1:cov21TRUE"] * splinepred[1] +
       fitted_model$cum[timepoint,"spline_age2:cov21TRUE"] * splinepred[2] +
       fitted_model$cum[timepoint,"spline_age3:cov21TRUE"] * splinepred[3]

     c20_cumhaz_age[i] <- cplot20_cumhaz_age
     c21_cumhaz_age[i] <- cplot21_cumhaz_age

     # covid*wlz-verblijf age-effect
     eeplot20_cumhaz_age <- fitted_model$cum[timepoint,"spline_age1:in_wlz_verblijf:cov20TRUE"] *
splinepred[1] +
       fitted_model$cum[timepoint,"spline_age2:in_wlz_verblijf:cov20TRUE"] * splinepred[2] +
       fitted_model$cum[timepoint,"spline_age3:in_wlz_verblijf:cov20TRUE"] * splinepred[3]

     eeplot21_cumhaz_age <- fitted_model$cum[timepoint,"spline_age1:in_wlz_verblijf:cov21TRUE"] *
splinepred[1] +
       fitted_model$cum[timepoint,"spline_age2:in_wlz_verblijf:cov21TRUE"] * splinepred[2] +
       fitted_model$cum[timepoint,"spline_age3:in_wlz_verblijf:cov21TRUE"] * splinepred[3]

     ee20_cumhaz_age[i] <- eeplot20_cumhaz_age
     ee21_cumhaz_age[i] <- eeplot21_cumhaz_age

   }

 df_plot <- data.frame(cbind(age = agevec, b_cumhaz_age, wlz_cumhaz_age, c20_cumhaz_age,
c21_cumhaz_age, ee20_cumhaz_age, ee21_cumhaz_age))
````
````

```
  return(df_plot)
}
```

Function to obtain the effects that do not depend on age at a given timepoint.
```{r}
GetNotAgeEffect <- function(agevec, timepoint, fitted_model){

  b_cumhaz_notage <- vector(length = length(agevec))
  wlz_cumhaz_notage <- vector(length = length(agevec))
  c20_cumhaz_notage <- vector(length = length(agevec))
  c21_cumhaz_notage <- vector(length = length(agevec))
  ee20_cumhaz_notage <- vector(length = length(agevec))
  ee21_cumhaz_notage <- vector(length = length(agevec))

  for (i in 1:length(agevec)){

    # background hazard not age-effect
    bplot_cumhaz_notage <- fitted_model$cum[timepoint,"(Intercept)"]

    b_cumhaz_notage[i] <- bplot_cumhaz_notage

  # wlz-verblijf hazard not age-effect
    wlzplot_cumhaz_notage <- fitted_model$cum[timepoint,"in_wlz_verblijf"]

    wlz_cumhaz_notage[i] <- wlzplot_cumhaz_notage

    # covid-19 hazard not age-effect
    cplot20_cumhaz_notage <- fitted_model$cum[timepoint,"cov20TRUE"]

    cplot21_cumhaz_notage <- fitted_model$cum[timepoint,"cov21TRUE"]

    c20_cumhaz_notage[i] <- cplot20_cumhaz_notage
    c21_cumhaz_notage[i] <- cplot21_cumhaz_notage

    # covid*wlz-verblijf not age-effect
    eeplot20_cumhaz_notage <- fitted_model$cum[timepoint,"in_wlz_verblijf:cov20TRUE"]

    eeplot21_cumhaz_notage <- fitted_model$cum[timepoint,"in_wlz_verblijf:cov21TRUE"]

    ee20_cumhaz_notage[i] <- eeplot20_cumhaz_notage
    ee21_cumhaz_notage[i] <- eeplot21_cumhaz_notage

    }

  df_plot <- data.frame(cbind(age = agevec, b_cumhaz_notage, wlz_cumhaz_notage, c20_cumhaz_notage,
c21_cumhaz_notage, ee20_cumhaz_notage, ee21_cumhaz_notage))
  return(df_plot)
}
```

The sum of the age-dependent and age-independent effect should be equal to the height of the respective hazard component at that particular timepoint (in this case: day 300).
```{r}
my_age <- 70:100

## TIMEPOINT 3 ONLY ##
# time-variable for 2020 and 2021
```

```
my_time <- 300
my_timepoint <- which.min(abs(fitadd_agespline_full_analysis_from70M$cum[,1] - my_time))

df_plotW_age <- GetAgeEffect(agevec = my_age, timepoint = my_timepoint, fitted_model =
fitadd_agespline_full_analysis_from70W, spline_age = spline_age)
df_plotW_notage <- GetNotAgeEffect(agevec = my_age, timepoint = my_timepoint, fitted_model =
fitadd_agespline_full_analysis_from70W)
df_plotW <- cbind(df_plotW_age, df_plotW_notage[-1])
df_plotW$sex <- "Women"

df_plotM_age <- GetAgeEffect(agevec = my_age, timepoint = my_timepoint, fitted_model =
fitadd_agespline_full_analysis_from70M, spline_age = spline_age)
df_plotM_notage <- GetNotAgeEffect(agevec = my_age, timepoint = my_timepoint, fitted_model =
fitadd_agespline_full_analysis_from70M)
df_plotM <- cbind(df_plotM_age, df_plotM_notage[-1])
df_plotM$sex <- "Men"

df_plotage_agespline2 <- rbind(df_plotW, df_plotM)

p.agespline2.total.effect.t300 <- df_plotage_agespline2 %>%
  ggplot(aes(x = age)) +
  geom_line(aes(y = b_cumhaz_age + b_cumhaz_notage, col = "blue")) +
  geom_line(aes(y = wlz_cumhaz_age + wlz_cumhaz_notage, col = "orange")) +
  geom_line(aes(y = c20_cumhaz_age + c20_cumhaz_notage, col = "purple")) +
  geom_line(aes(y = c21_cumhaz_age + c21_cumhaz_notage, col = "purple4")) +
  geom_line(aes(y = ee20_cumhaz_age + ee20_cumhaz_notage, col = "red")) +
  geom_line(aes(y = ee21_cumhaz_age + ee21_cumhaz_notage, col = "red4")) +
  facet_grid(~sex) +
  labs(x = "Age", y = "Cumulative hazard", title = "Total (splines, knots at 80, 90), time = day 300") +
  scale_color_manual(name = "Mortality components",
                values = c("orange" = "orange", "blue" = "blue", "purple" = "purple", "purple4" = "purple4",
"red" = "red", "red4" = "red4"),
                labels = c("blue" = "Background", "orange" = "Excess: care home stay", "purple"= "Excess:
Covid-19 pandemic (2020)","purple4"="Excess: Covid-19 pandemic (2021)", "red"= "Excess: excess
(2020)", "red4"= "Excess: excess (2021)"))
p.agespline2.total.effect.t300
ggsave(p.agespline2.total.effect.t300, file =
"../figures/p.agespline2.total.effect.t300.full_analysis_from70.png", width=10,height=4,dpi=300)
```

We can also only plot the age-related effect, which is slightly more informative if the aim is to investigate the shape of the age-effect per hazard component.

```{r}
my_age <- 70:100

## TIMEPOINT 1 ##
# time-variable for 2020 and 2021

my_time <- 100
my_timepoint <- which.min(abs(fitadd_agespline_full_analysis_from70M$cum[,1] - my_time))

df_plotW <- GetAgeEffect(agevec = my_age, timepoint = my_timepoint, fitted_model =
fitadd_agespline_full_analysis_from70W, spline_age = spline_age)
df_plotW$sex <- "Women"
df_plotM <- GetAgeEffect(agevec = my_age, timepoint = my_timepoint, fitted_model =
fitadd_agespline_full_analysis_from70M, spline_age = spline_age)
df_plotM$sex <- "Men"

df_plotage_agespline2_t100 <- rbind(df_plotW, df_plotM)
```

```
p.agespline.age.effect.t100 <- df_plotage_agespline2_t100 %>%
  ggplot(aes(x = age)) +
  geom_line(aes(y = b_cumhaz_age, col = "blue")) +
  geom_line(aes(y = wlz_cumhaz_age, col = "orange")) +
  geom_line(aes(y = c20_cumhaz_age, col = "purple")) +
  geom_line(aes(y = c21_cumhaz_age, col = "purple4")) +
  geom_line(aes(y = ee20_cumhaz_age, col = "red")) +
  geom_line(aes(y = ee21_cumhaz_age, col = "red4")) +
  facet_grid(~sex) +
  labs(x = "Age", y = "Cumulative hazard", title = "Age effect (splines, knots at 80, 90), time = day 100") +
  scale_color_manual(name = "Mortality components",
                values = c("orange" = "orange", "blue" = "blue", "purple" = "purple", "purple4" = "purple4",
"red" = "red", "red4" = "red4"),
                labels = c("blue" = "Background", "orange" = "Excess: care home stay", "purple"= "Excess:
Covid-19 pandemic (2020)","purple4"="Excess: Covid-19 pandemic (2021)", "red"= "Excess: excess
(2020)", "red4"= "Excess: excess (2021)"))

p.agespline.age.effect.t100
ggsave(p.agespline.age.effect.t100, file = "../figures/p.agespline.age.effect.t100.full_analysis_from70.png",
width=10,height=4,dpi=300)


## TIMEPOINT 2 ##
# time-variable for 2020 and 2021
my_time <- 200
my_timepoint <- which.min(abs(fitadd_agespline_full_analysis_from70M$cum[,1] - my_time))

df_plotW <- GetAgeEffect(agevec = my_age, timepoint = my_timepoint, fitted_model =
fitadd_agespline_full_analysis_from70W, spline_age = spline_age)
df_plotW$sex <- "Women"
df_plotM <- GetAgeEffect(agevec = my_age, timepoint = my_timepoint, fitted_model =
fitadd_agespline_full_analysis_from70M, spline_age = spline_age)
df_plotM$sex <- "Men"

df_plotage_agespline2_t200  <- rbind(df_plotW, df_plotM)

p.agespline.age.effect.t200 <- df_plotage_agespline2_t200 %>%
  ggplot(aes(x = age)) +
  geom_line(aes(y = b_cumhaz_age, col = "blue")) +
  geom_line(aes(y = wlz_cumhaz_age, col = "orange")) +
  geom_line(aes(y = c20_cumhaz_age, col = "purple")) +
  geom_line(aes(y = c21_cumhaz_age, col = "purple4")) +
  geom_line(aes(y = ee20_cumhaz_age, col = "red")) +
  geom_line(aes(y = ee21_cumhaz_age, col = "red4")) +
  facet_grid(~sex) +
  labs(x = "Age", y = "Cumulative hazard", title = "Age effect (splines, knots at 80, 90), time = day 200") +
  scale_color_manual(name = "Mortality components",
                values = c("orange" = "orange", "blue" = "blue", "purple" = "purple", "purple4" = "purple4",
"red" = "red", "red4" = "red4"),
                labels = c("blue" = "Background", "orange" = "Excess: care home stay", "purple"= "Excess:
Covid-19 pandemic (2020)","purple4"="Excess: Covid-19 pandemic (2021)", "red"= "Excess: excess
(2020)", "red4"= "Excess: excess (2021)"))

p.agespline.age.effect.t200
ggsave(p.agespline.age.effect.t200, file = "../figures/p.agespline.age.effect.t200.full_analysis_from70.png",
width=10,height=4,dpi=300)

## TIMEPOINT 3 ##
```

```
# time-variable for 2020 and 2021
my_time <- 300
my_timepoint <- which.min(abs(fitadd_agespline_full_analysis_from70M$cum[,1] - my_time))

df_plotW <- GetAgeEffect(agevec = my_age, timepoint = my_timepoint, fitted_model =
fitadd_agespline_full_analysis_from70W, spline_age = spline_age)
df_plotW$sex <- "Women"
df_plotM <- GetAgeEffect(agevec = my_age, timepoint = my_timepoint, fitted_model =
fitadd_agespline_full_analysis_from70M, spline_age = spline_age)
df_plotM$sex <- "Men"

df_plotage_agespline2_t300 <- rbind(df_plotW, df_plotM)

p.agespline.age.effect.t300 <- df_plotage_agespline2_t300 %>%
  ggplot(aes(x = age)) +
  geom_line(aes(y = b_cumhaz_age, col = "blue")) +
  geom_line(aes(y = wlz_cumhaz_age, col = "orange")) +
  geom_line(aes(y = c20_cumhaz_age, col = "purple")) +
  geom_line(aes(y = c21_cumhaz_age, col = "purple4")) +
  geom_line(aes(y = ee20_cumhaz_age, col = "red")) +
  geom_line(aes(y = ee21_cumhaz_age, col = "red4")) +
  facet_grid(~sex) +
  labs(x = "Age", y = "Cumulative hazard", title = "Age effect (splines, knots at 80, 90), time = day 300") +
  scale_color_manual(name = "Mortality components",
                values = c("orange" = "orange", "blue" = "blue", "purple" = "purple", "purple4" = "purple4",
"red" = "red", "red4" = "red4"),
                labels = c("blue" = "Background", "orange" = "Excess: care home stay", "purple"= "Excess:
Covid-19 pandemic (2020)","purple4"="Excess: Covid-19 pandemic (2021)", "red"= "Excess: excess
(2020)", "red4"= "Excess: excess (2021)"))

p.agespline.age.effect.t300
ggsave(p.agespline.age.effect.t300, file = "../figures/p.agespline.age.effect.t300.full_analysis_from70.png",
width=10,height=4,dpi=300)
```

Model 3: age effect splines (knots 85)

Now we consider a model with just one knot (and 2 boundary knots), located at age 85.

```{r}
my_knots <- c(85)
my_boundary_knots <- c(70, 100)

spline_age <- ns(data_use_subset$age_floor, knots = my_knots, Boundary.knots = my_boundary_knots)
```

M: Fit model

```{r, eval = F}
spline_age <- ns(data_use_subset$age_floor, knots = my_knots, Boundary.knots = my_boundary_knots)
spline_age <- spline_age[data_use_subset$sex == "male",] # temporary, to get correct covariate names in
model
gc()

fitadd_agespline_full_analysis_from70bM <- aalen(Surv(entry, srv, srv_s) ~
                    spline_age +
            in_wlz_verblijf + in_wlz_verblijf:spline_age +
            cov20 + cov20:spline_age +
            cov21 + cov21:spline_age +
            cov20:in_wlz_verblijf + cov20:spline_age:in_wlz_verblijf +
```

            cov21:in_wlz_verblijf + cov21:spline_age:in_wlz_verblijf,
              data = data_use_subset[data_use_subset$sex == "male",], robust = 0)

save(fitadd_agespline_full_analysis_from70bM, file =
"../objects/fitadd_agespline_full_analysis_from70bM.RData")
spline_age <- ns(data_use_subset$age_floor, knots = my_knots, Boundary.knots = my_boundary_knots)
```

## W: Fit model
```{r, eval = F}
spline_age <- ns(data_use_subset$age_floor, knots = my_knots, Boundary.knots = my_boundary_knots)
spline_age <- spline_age[data_use_subset$sex == "female",] # temporary, to get correct covariate names in model
gc()

fitadd_agespline_full_analysis_from70bW <- aalen(Surv(entry, srv, srv_s) ~
                  spline_age +
            in_wlz_verblijf + in_wlz_verblijf:spline_age +
            cov20 + cov20:spline_age +
            cov21 + cov21:spline_age +
            cov20:in_wlz_verblijf + cov20:spline_age:in_wlz_verblijf +
            cov21:in_wlz_verblijf + cov21:spline_age:in_wlz_verblijf,
              data = data_use_subset[data_use_subset$sex == "female",], robust = 0)


save(fitadd_agespline_full_analysis_from70bW, file =
"../objects/fitadd_agespline_full_analysis_from70bW.RData")
spline_age <- ns(data_use_subset$age_floor, knots = my_knots, Boundary.knots = my_boundary_knots)
```

Plot results

```{r}
gc()

load("H:/DECIM_Marije/excess_excess/additive_hazards/objects/fitadd_agespline_full_analysis_from70bM.RData")
load("H:/DECIM_Marije/excess_excess/additive_hazards/objects/fitadd_agespline_full_analysis_from70bW.RData")
```

```{r}
GetHazards <- function(agevar, fitted_model, splinepred){

  # time-variable for 2020 and 2021
  tplot20 <- fitted_model$cum[,1]
  tplot21 <- tplot20 + max(tplot20) - 1 # -1 because Jan 1, 2021 is the 367th day of my model (not the 368th)
  tplot <- c(tplot20[-1], tplot21[-1]) # first element not counted because that is 'day 0' when I let everyone enter but no one die
  tplot <- tplot[-length(tplot)] # remove last element because 2021 only has 365 days

  #   background hazard: intercept + sex + age + age:sex
  bplot20 <- fitted_model$cum[,"(Intercept)"] +
   fitted_model$cum[,"spline_age1"] * splinepred[1] +
   fitted_model$cum[,"spline_age2"] * splinepred[2]

  bplot21 <- bplot20 + tail(bplot20, 1)

````
  bplot <- c(bplot20[-1], bplot21[-1])
  bplot <- bplot[-length(bplot)]

  #   wlz-verblijf hazard: wlz-main, wlz:age, wlz:sex, wlz:age:sex
  wlzplot20 <- fitted_model$cum[,"in_wlz_verblijf"] +
   fitted_model$cum[,"spline_age1:in_wlz_verblijf"] * splinepred[1] +
   fitted_model$cum[,"spline_age2:in_wlz_verblijf"] * splinepred[2]

  wlzplot21 <- wlzplot20 + tail(wlzplot20, 1)
  wlzplot <- c(wlzplot20[-1], wlzplot21[-1])
  wlzplot <- wlzplot[-length(wlzplot)]

  #   covid-19 hazard: covid20-main, covid20:age_cat, covid20:sex, covid20:age_cat:sex (same for
covid21)
  cplot20 <- fitted_model$cum[,"cov20TRUE"] +
   fitted_model$cum[,"spline_age1:cov20TRUE"] * splinepred[1] +
   fitted_model$cum[,"spline_age2:cov20TRUE"] * splinepred[2]

  c21 <- fitted_model$cum[,"cov21TRUE"] +
   fitted_model$cum[,"spline_age1:cov21TRUE"] * splinepred[1] +
   fitted_model$cum[,"spline_age2:cov21TRUE"] * splinepred[2]

  cplot21 <- c21 + tail(cplot20, n = 1)
  cplot <- c(cplot20[-1], cplot21[-1])
  cplot <- cplot[-length(cplot)]

  #   covid*wlz-verblijf hazard: covid20:wlz, covid20:wlz:age, covid20:wlz:sex, covid20:wlz:age:sex (same
for covid21)
  eeplot20 <- fitted_model$cum[,"in_wlz_verblijf:cov20TRUE"] +
   fitted_model$cum[,"spline_age1:in_wlz_verblijf:cov20TRUE"] * splinepred[1] +
   fitted_model$cum[,"spline_age2:in_wlz_verblijf:cov20TRUE"] * splinepred[2]

  ee21 <- fitted_model$cum[,"in_wlz_verblijf:cov21TRUE"] +
   fitted_model$cum[,"spline_age1:in_wlz_verblijf:cov21TRUE"] * splinepred[1] +
   fitted_model$cum[,"spline_age2:in_wlz_verblijf:cov21TRUE"] * splinepred[2]

  eeplot21 <- ee21 + tail(eeplot20, n = 1)
  eeplot <- c(eeplot20[-1], eeplot21[-1])
  eeplot <- eeplot[-length(eeplot)]

  df_plot <- data.frame(tplot = tplot,
                        bplot = bplot,
                        wlzplot = wlzplot,
                        cplot = cplot,
                        eeplot = eeplot,
                        age = agevar)

  return(df_plot)
}
```

```{r}
my_agevars = c(75, 85, 95)

## Men ##

df_plothaz_agespline1_listM <- list()

for (i in 1:length(my_agevars)){
````

~~~
  my_splinepred <- predict(spline_age, my_agevars[i])[1,]
  df_plothaz_agespline1 <- GetHazards(agevar = my_agevars[i], fitted_model =
fitadd_agespline_full_analysis_from70bM, splinepred = my_splinepred)
  df_plothaz_agespline1_listM[[i]] <- df_plothaz_agespline1
}

df_plothaz_agespline1_list_coarsenedM <- lapply(df_plothaz_agespline1_listM, Coarsen, n_timepoints =
2000)
df_plothaz_agespline1M <- list_rbind(df_plothaz_agespline1_list_coarsenedM)

df_plothaz_agespline1M$sex <- "Men"

## Women ##

df_plothaz_agespline1_listW <- list()

for (i in 1:length(my_agevars)){

  my_splinepred <- predict(spline_age, my_agevars[i])[1,]
  df_plothaz_agespline1 <- GetHazards(agevar = my_agevars[i], fitted_model =
fitadd_agespline_full_analysis_from70bW, splinepred = my_splinepred)
  df_plothaz_agespline1_listW[[i]] <- df_plothaz_agespline1
}

df_plothaz_agespline1_list_coarsenedW <- lapply(df_plothaz_agespline1_listW, Coarsen, n_timepoints =
2000)
df_plothaz_agespline1W <- list_rbind(df_plothaz_agespline1_list_coarsenedW)

df_plothaz_agespline1W$sex <- "Women"

df_plothaz_agespline1 <- rbind(df_plothaz_agespline1M, df_plothaz_agespline1W)

p.allhaz.grid.agespline <- df_plothaz_agespline1 %>%
  ggplot(aes(x = tplot)) +
  geom_line(aes(y = bplot, col = "blue")) +
  geom_line(aes(y = wlzplot, col = "orange")) +
  geom_line(aes(y = cplot, col = "purple")) +
  geom_line(aes(y = eeplot, col = "red")) +
  labs(x = "Days (2020 + 2021)", y = "Cumulative hazard", title = "Age with splines (knot at 85)") +
  scale_color_manual(name = "Mortality components", values = c("orange" = "orange", "blue" = "blue",
"purple" = "purple", "red" = "red"), labels = c("Background", "Excess: care home stay", "Excess: Covid-19
pandemic", "Excess: excess")) +
  facet_grid(sex~age) +
  coord_cartesian(ylim = c(-0.1, 0.9))
p.allhaz.grid.agespline

ggsave(p.allhaz.grid.agespline, file = "../figures/p.allhaz.grid.agespline.full_analysis_from70b.png",
width=10,height=4,dpi=300)
```

## Plot age effect

```{r}
GetAgeEffect <- function(sexvar, agevec, timepoint, fitted_model, spline_age){

  b_cumhaz_age <- vector(length = length(agevec))
  wlz_cumhaz_age <- vector(length = length(agevec))
  c20_cumhaz_age <- vector(length = length(agevec))
~~~

````
  c21_cumhaz_age <- vector(length = length(agevec))
  ee20_cumhaz_age <- vector(length = length(agevec))
  ee21_cumhaz_age <- vector(length = length(agevec))

  for (i in 1:length(agevec)){

    splinepred <- predict(spline_age, agevec[i])[1,]

    # background hazard age-effect
    bplot_cumhaz_age <- fitted_model$cum[timepoint,"spline_age1"] * splinepred[1] +
      fitted_model$cum[timepoint,"spline_age2"] * splinepred[2]

     b_cumhaz_age[i] <- bplot_cumhaz_age

    # wlz-verblijf hazard age-effect
      wlzplot_cumhaz_age <- fitted_model$cum[timepoint,"spline_age1:in_wlz_verblijf"] * splinepred[1] +
        fitted_model$cum[timepoint,"spline_age2:in_wlz_verblijf"] * splinepred[2]

      wlz_cumhaz_age[i] <- wlzplot_cumhaz_age

      # covid-19 hazard age-effect
      cplot20_cumhaz_age <- fitted_model$cum[timepoint,"spline_age1:cov20TRUE"] * splinepred[1] +
        fitted_model$cum[timepoint,"spline_age2:cov20TRUE"] * splinepred[2]

      cplot21_cumhaz_age <- fitted_model$cum[timepoint,"spline_age1:cov21TRUE"] * splinepred[1] +
        fitted_model$cum[timepoint,"spline_age2:cov21TRUE"] * splinepred[2]

      c20_cumhaz_age[i] <- cplot20_cumhaz_age
      c21_cumhaz_age[i] <- cplot21_cumhaz_age

      # covid*wlz-verblijf hazard
      eeplot20_cumhaz_age <- fitted_model$cum[timepoint,"spline_age1:in_wlz_verblijf:cov20TRUE"] *
splinepred[1] +
        fitted_model$cum[timepoint,"spline_age2:in_wlz_verblijf:cov20TRUE"] * splinepred[2]

      eeplot21_cumhaz_age <- fitted_model$cum[timepoint,"spline_age1:in_wlz_verblijf:cov21TRUE"] *
splinepred[1] +
        fitted_model$cum[timepoint,"spline_age2:in_wlz_verblijf:cov21TRUE"] * splinepred[2]

      ee20_cumhaz_age[i] <- eeplot20_cumhaz_age
      ee21_cumhaz_age[i] <- eeplot21_cumhaz_age

    }

  df_plot <- data.frame(cbind(age = agevec, b_cumhaz_age, wlz_cumhaz_age, c20_cumhaz_age,
c21_cumhaz_age, ee20_cumhaz_age, ee21_cumhaz_age))
  return(df_plot)
}
```

```{r}
my_age <- 70:100

## TIMEPOINT 1 ##
# time-variable for 2020 and 2021

my_time <- 100
my_timepoint <- which.min(abs(fitadd_agespline_full_analysis_from70bM$cum[,1] - my_time))
````

```
df_plotW <- GetAgeEffect(agevec = my_age, timepoint = my_timepoint, fitted_model =
fitadd_agespline_full_analysis_from70bW, spline_age = spline_age)
df_plotW$sex <- "Women"
df_plotM <- GetAgeEffect(agevec = my_age, timepoint = my_timepoint, fitted_model =
fitadd_agespline_full_analysis_from70bM, spline_age = spline_age)
df_plotM$sex <- "Men"

df_plotage_agespline1_t100 <- rbind(df_plotW, df_plotM)

p.agespline.age.effect.t100 <- df_plotage_agespline1_t100 %>%
  ggplot(aes(x = age)) +
  geom_line(aes(y = b_cumhaz_age, col = "blue")) +
  geom_line(aes(y = wlz_cumhaz_age, col = "orange")) +
  geom_line(aes(y = c20_cumhaz_age, col = "purple")) +
  geom_line(aes(y = c21_cumhaz_age, col = "purple4")) +
  geom_line(aes(y = ee20_cumhaz_age, col = "red")) +
  geom_line(aes(y = ee21_cumhaz_age, col = "red4")) +
  facet_grid(~sex) +
  labs(x = "Age", y = "Cumulative hazard", title = "Age effect (splines, knot at 85), time = day 100") +
  scale_color_manual(name = "Mortality components",
                values = c("orange" = "orange", "blue" = "blue", "purple" = "purple", "purple4" = "purple4",
"red" = "red", "red4" = "red4"),
                labels = c("blue" = "Background", "orange" = "Excess: care home stay", "purple"= "Excess:
Covid-19 pandemic (2020)","purple4"="Excess: Covid-19 pandemic (2021)", "red"= "Excess: excess
(2020)", "red4"= "Excess: excess (2021)"))

p.agespline.age.effect.t100
ggsave(p.agespline.age.effect.t100, file = "../figures/p.agespline.age.effect.t100.full_analysis_from70b.png",
width=10,height=4,dpi=300)


## TIMEPOINT 2 ##
# time-variable for 2020 and 2021
my_time <- 200
my_timepoint <- which.min(abs(fitadd_agespline_full_analysis_from70bM$cum[,1] - my_time))

df_plotW <- GetAgeEffect(agevec = my_age, timepoint = my_timepoint, fitted_model =
fitadd_agespline_full_analysis_from70bW, spline_age = spline_age)
df_plotW$sex <- "Women"
df_plotM <- GetAgeEffect(agevec = my_age, timepoint = my_timepoint, fitted_model =
fitadd_agespline_full_analysis_from70bM, spline_age = spline_age)
df_plotM$sex <- "Men"

df_plotage_agespline1_t200 <- rbind(df_plotW, df_plotM)

p.agespline.age.effect.t200 <- df_plotage_agespline1_t200 %>%
  ggplot(aes(x = age)) +
  geom_line(aes(y = b_cumhaz_age, col = "blue")) +
  geom_line(aes(y = wlz_cumhaz_age, col = "orange")) +
  geom_line(aes(y = c20_cumhaz_age, col = "purple")) +
  geom_line(aes(y = c21_cumhaz_age, col = "purple4")) +
  geom_line(aes(y = ee20_cumhaz_age, col = "red")) +
  geom_line(aes(y = ee21_cumhaz_age, col = "red4")) +
  facet_grid(~sex) +
  labs(x = "Age", y = "Cumulative hazard", title = "Age effect (splines, knot at 85), time = day 200") +
  scale_color_manual(name = "Mortality components",
                values = c("orange" = "orange", "blue" = "blue", "purple" = "purple", "purple4" = "purple4",
"red" = "red", "red4" = "red4"),
```

                 labels = c("blue" = "Background", "orange" = "Excess: care home stay", "purple"= "Excess: Covid-19 pandemic (2020)","purple4"="Excess: Covid-19 pandemic (2021)", "red"= "Excess: excess (2020)", "red4"= "Excess: excess (2021)"))

p.agespline.age.effect.t200
ggsave(p.agespline.age.effect.t200, file = "../figures/p.agespline.age.effect.t200.full_analysis_from70b.png", width=10,height=4,dpi=300)

TIMEPOINT 3
# time-variable for 2020 and 2021
my_time <- 300
my_timepoint <- which.min(abs(fitadd_agespline_full_analysis_from70bM$cum[,1] - my_time))

df_plotW <- GetAgeEffect(sexvar = 0, agevec = my_age, timepoint = my_timepoint, fitted_model = fitadd_agespline_full_analysis_from70bW, spline_age = spline_age)
df_plotW$sex <- "Women"
df_plotM <- GetAgeEffect(sexvar = 1, agevec = my_age, timepoint = my_timepoint, fitted_model = fitadd_agespline_full_analysis_from70bM, spline_age = spline_age)
df_plotM$sex <- "Men"

df_plotage_agespline1_t300 <- rbind(df_plotW, df_plotM)

p.agespline.age.effect.t300 <- df_plotage_agespline1_t300 %>%
  ggplot(aes(x = age)) +
  geom_line(aes(y = b_cumhaz_age, col = "blue")) +
  geom_line(aes(y = wlz_cumhaz_age, col = "orange")) +
  geom_line(aes(y = c20_cumhaz_age, col = "purple")) +
  geom_line(aes(y = c21_cumhaz_age, col = "purple4")) +
  geom_line(aes(y = ee20_cumhaz_age, col = "red")) +
  geom_line(aes(y = ee21_cumhaz_age, col = "red4")) +
  facet_grid(~sex) +
  labs(x = "Age", y = "Cumulative hazard", title = "Age effect (splines, knot at 85), time = day 300") +
  scale_color_manual(name = "Mortality components",
                 values = c("orange" = "orange", "blue" = "blue", "purple" = "purple", "purple4" = "purple4", "red" = "red", "red4" = "red4"),
                 labels = c("blue" = "Background", "orange" = "Excess: care home stay", "purple"= "Excess: Covid-19 pandemic (2020)","purple4"="Excess: Covid-19 pandemic (2021)", "red"= "Excess: excess (2020)", "red4"= "Excess: excess (2021)"))

p.agespline.age.effect.t300
ggsave(p.agespline.age.effect.t300, file = "../figures/p.agespline.age.effect.t300.full_analysis_from70b.png", width=10,height=4,dpi=300)
```

# Model 4: age effect splines (knots 75, 85, 95)

Now we consider a model with 3 knots (and 2 boundary knots).

```{r}
my_knots <- c(75, 85, 95)
my_boundary_knots <- c(70, 100)

spline_age <- ns(data_use_subset$age_floor, knots = my_knots, Boundary.knots = my_boundary_knots)
```

M: Fit model
```{r, eval = F}
spline_age <- ns(data_use_subset$age_floor, knots = my_knots, Boundary.knots = my_boundary_knots)

spline_age <- spline_age[data_use_subset$sex == "male",] # temporary, to get correct covariate names in model
gc()

fitadd_agespline_full_analysis_from70cM <- aalen(Surv(entry, srv, srv_s) ~
                     spline_age +
            in_wlz_verblijf + in_wlz_verblijf:spline_age +
            cov20 + cov20:spline_age +
            cov21 + cov21:spline_age +
            cov20:in_wlz_verblijf + cov20:spline_age:in_wlz_verblijf +
            cov21:in_wlz_verblijf + cov21:spline_age:in_wlz_verblijf,
              data = data_use_subset[data_use_subset$sex == "male",], robust = 0)


save(fitadd_agespline_full_analysis_from70cM, file =
"../objects/fitadd_agespline_full_analysis_from70cM.RData")
spline_age <- ns(data_use_subset$age_floor, knots = my_knots, Boundary.knots = my_boundary_knots)
```

W: Fit model
```{r, eval = F}
spline_age <- ns(data_use_subset$age_floor, knots = my_knots, Boundary.knots = my_boundary_knots)
spline_age <- spline_age[data_use_subset$sex == "female",] # temporary, to get correct covariate names in model
gc()

fitadd_agespline_full_analysis_from70cW <- aalen(Surv(entry, srv, srv_s) ~
                     spline_age +
            in_wlz_verblijf + in_wlz_verblijf:spline_age +
            cov20 + cov20:spline_age +
            cov21 + cov21:spline_age +
            cov20:in_wlz_verblijf + cov20:spline_age:in_wlz_verblijf +
            cov21:in_wlz_verblijf + cov21:spline_age:in_wlz_verblijf,
              data = data_use_subset[data_use_subset$sex == "female",], robust = 0)


save(fitadd_agespline_full_analysis_from70cW, file =
"../objects/fitadd_agespline_full_analysis_from70cW.RData")
spline_age <- ns(data_use_subset$age_floor, knots = my_knots, Boundary.knots = my_boundary_knots)
```

Plot results

```{r}
gc()

load("H:/DECIM_Marije/excess_excess/additive_hazards/objects/fitadd_agespline_full_analysis_from70cM.RData")
load("H:/DECIM_Marije/excess_excess/additive_hazards/objects/fitadd_agespline_full_analysis_from70cW.RData")
```

```{r}
GetHazards <- function(agevar, fitted_model, splinepred){

  # time-variable for 2020 and 2021
  tplot20 <- fitted_model$cum[,1]
  tplot21 <- tplot20 + max(tplot20) - 1 # -1 because Jan 1, 2021 is the 367th day of my model (not the 368th)

```
  tplot <- c(tplot20[-1], tplot21[-1]) # first element not counted because that is 'day 0' when I let everyone
enter but no one die
  tplot <- tplot[-length(tplot)] # remove last element because 2021 only has 365 days

  #   background hazard: intercept + sex + age + age:sex
  bplot20 <- fitted_model$cum[,"(Intercept)"] +
    fitted_model$cum[,"spline_age1"] * splinepred[1] +
    fitted_model$cum[,"spline_age2"] * splinepred[2] +
    fitted_model$cum[,"spline_age3"] * splinepred[3] +
    fitted_model$cum[,"spline_age4"] * splinepred[4]

  bplot21 <- bplot20 + tail(bplot20, 1)
  bplot <- c(bplot20[-1], bplot21[-1])
  bplot <- bplot[-length(bplot)]

  #   wlz-verblijf hazard: wlz-main, wlz:age, wlz:sex, wlz:age:sex
  wlzplot20 <- fitted_model$cum[,"in_wlz_verblijf"] +
    fitted_model$cum[,"spline_age1:in_wlz_verblijf"] * splinepred[1] +
    fitted_model$cum[,"spline_age2:in_wlz_verblijf"] * splinepred[2] +
    fitted_model$cum[,"spline_age3:in_wlz_verblijf"] * splinepred[3] +
    fitted_model$cum[,"spline_age4:in_wlz_verblijf"] * splinepred[4]

  wlzplot21 <- wlzplot20 + tail(wlzplot20, 1)
  wlzplot <- c(wlzplot20[-1], wlzplot21[-1])
  wlzplot <- wlzplot[-length(wlzplot)]

  #   covid-19 hazard: covid20-main, covid20:age_cat, covid20:sex, covid20:age_cat:sex (same for
covid21)
  cplot20 <- fitted_model$cum[,"cov20TRUE"] +
    fitted_model$cum[,"spline_age1:cov20TRUE"] * splinepred[1] +
    fitted_model$cum[,"spline_age2:cov20TRUE"] * splinepred[2] +
    fitted_model$cum[,"spline_age3:cov20TRUE"] * splinepred[3] +
    fitted_model$cum[,"spline_age4:cov20TRUE"] * splinepred[4]

  c21 <- fitted_model$cum[,"cov21TRUE"] +
    fitted_model$cum[,"spline_age1:cov21TRUE"] * splinepred[1] +
    fitted_model$cum[,"spline_age2:cov21TRUE"] * splinepred[2] +
    fitted_model$cum[,"spline_age3:cov21TRUE"] * splinepred[3] +
    fitted_model$cum[,"spline_age4:cov21TRUE"] * splinepred[4]

  cplot21 <- c21 + tail(cplot20, n = 1)
  cplot <- c(cplot20[-1], cplot21[-1])
  cplot <- cplot[-length(cplot)]

  #   covid*wlz-verblijf hazard: covid20:wlz, covid20:wlz:age, covid20:wlz:sex, covid20:wlz:age:sex (same
for covid21)
  eeplot20 <- fitted_model$cum[,"in_wlz_verblijf:cov20TRUE"] +
    fitted_model$cum[,"spline_age1:in_wlz_verblijf:cov20TRUE"] * splinepred[1] +
    fitted_model$cum[,"spline_age2:in_wlz_verblijf:cov20TRUE"] * splinepred[2] +
    fitted_model$cum[,"spline_age3:in_wlz_verblijf:cov20TRUE"] * splinepred[3] +
    fitted_model$cum[,"spline_age4:in_wlz_verblijf:cov20TRUE"] * splinepred[4]

  ee21 <- fitted_model$cum[,"in_wlz_verblijf:cov21TRUE"] +
    fitted_model$cum[,"spline_age1:in_wlz_verblijf:cov21TRUE"] * splinepred[1] +
    fitted_model$cum[,"spline_age2:in_wlz_verblijf:cov21TRUE"] * splinepred[2] +
    fitted_model$cum[,"spline_age3:in_wlz_verblijf:cov21TRUE"] * splinepred[3] +
    fitted_model$cum[,"spline_age4:in_wlz_verblijf:cov21TRUE"] * splinepred[4]

  eeplot21 <- ee21 + tail(eeplot20, n = 1)
```

```
    eeplot <- c(eeplot20[-1], eeplot21[-1])
    eeplot <- eeplot[-length(eeplot)]

    df_plot <- data.frame(tplot = tplot,
                          bplot = bplot,
                          wlzplot = wlzplot,
                          cplot = cplot,
                          eeplot = eeplot,
                          age = agevar)

    return(df_plot)
}
```

```{r}
my_agevars = c(75, 85, 95)

## Men ##

df_plothaz_agespline3_listM <- list()

for (i in 1:length(my_agevars)){

  my_splinepred <- predict(spline_age, my_agevars[i])[1,]
  df_plothaz_agespline3 <- GetHazards(agevar = my_agevars[i], fitted_model =
fitadd_agespline_full_analysis_from70cM, splinepred = my_splinepred)
  df_plothaz_agespline3_listM[[i]] <- df_plothaz_agespline3
}

df_plothaz_agespline3_list_coarsenedM <- lapply(df_plothaz_agespline3_listM, Coarsen, n_timepoints =
2000)
df_plothaz_agespline3M <- list_rbind(df_plothaz_agespline3_list_coarsenedM)

df_plothaz_agespline3M$sex <- "Men"

## Women ##

df_plothaz_agespline3_listW <- list()

for (i in 1:length(my_agevars)){

  my_splinepred <- predict(spline_age, my_agevars[i])[1,]
  df_plothaz_agespline3 <- GetHazards(agevar = my_agevars[i], fitted_model =
fitadd_agespline_full_analysis_from70cW, splinepred = my_splinepred)
  df_plothaz_agespline3_listW[[i]] <- df_plothaz_agespline3
}

df_plothaz_agespline3_list_coarsenedW <- lapply(df_plothaz_agespline3_listW, Coarsen, n_timepoints =
2000)
df_plothaz_agespline3W <- list_rbind(df_plothaz_agespline3_list_coarsenedW)

df_plothaz_agespline3W$sex <- "Women"

df_plothaz_agespline3 <- rbind(df_plothaz_agespline3M, df_plothaz_agespline3W)

p.allhaz.grid.agespline <- df_plothaz_agespline3 %>%
  ggplot(aes(x = tplot)) +
  geom_line(aes(y = bplot, col = "blue")) +
  geom_line(aes(y = wlzplot, col = "orange")) +
```

```
  geom_line(aes(y = cplot, col = "purple")) +
  geom_line(aes(y = eeplot, col = "red")) +
  labs(x = "Days (2020 + 2021)", y = "Cumulative hazard", title = "Age with splines (knots at 75, 85, 95)") +
  scale_color_manual(name = "Mortality components", values = c("orange" = "orange", "blue" = "blue",
"purple" = "purple", "red" = "red"), labels = c("Background", "Excess: care home stay", "Excess: Covid-19
pandemic", "Excess: excess")) +
  facet_grid(sex~age) +
  coord_cartesian(ylim = c(-0.1, 0.9))
p.allhaz.grid.agespline

ggsave(p.allhaz.grid.agespline, file = "../figures/p.allhaz.grid.agespline.full_analysis_from70c.png",
width=10,height=4,dpi=300)
```

Plot age effect

```{r}
GetAgeEffect <- function(sexvar, agevec, timepoint, fitted_model, spline_age){

  b_cumhaz_age <- vector(length = length(agevec))
  wlz_cumhaz_age <- vector(length = length(agevec))
  c20_cumhaz_age <- vector(length = length(agevec))
  c21_cumhaz_age <- vector(length = length(agevec))
  ee20_cumhaz_age <- vector(length = length(agevec))
  ee21_cumhaz_age <- vector(length = length(agevec))

  for (i in 1:length(agevec)){

    splinepred <- predict(spline_age, agevec[i])[1,]

    # background hazard age-effect
    bplot_cumhaz_age <- fitted_model$cum[timepoint,"spline_age1"] * splinepred[1] +
      fitted_model$cum[timepoint,"spline_age2"] * splinepred[2] +
      fitted_model$cum[timepoint,"spline_age3"] * splinepred[3] +
      fitted_model$cum[timepoint,"spline_age4"] * splinepred[4]

     b_cumhaz_age[i] <- bplot_cumhaz_age

    # wlz-verblijf hazard age-effect
      wlzplot_cumhaz_age <- fitted_model$cum[timepoint,"spline_age1:in_wlz_verblijf"] * splinepred[1] +
        fitted_model$cum[timepoint,"spline_age2:in_wlz_verblijf"] * splinepred[2] +
        fitted_model$cum[timepoint,"spline_age3:in_wlz_verblijf"] * splinepred[3] +
        fitted_model$cum[timepoint,"spline_age4:in_wlz_verblijf"] * splinepred[4]

      wlz_cumhaz_age[i] <- wlzplot_cumhaz_age

      # covid-19 hazard age-effect
      cplot20_cumhaz_age <- fitted_model$cum[timepoint,"spline_age1:cov20TRUE"] * splinepred[1] +
        fitted_model$cum[timepoint,"spline_age2:cov20TRUE"] * splinepred[2] +
        fitted_model$cum[timepoint,"spline_age3:cov20TRUE"] * splinepred[3] +
        fitted_model$cum[timepoint,"spline_age4:cov20TRUE"] * splinepred[4]

      cplot21_cumhaz_age <- fitted_model$cum[timepoint,"spline_age1:cov21TRUE"] * splinepred[1] +
        fitted_model$cum[timepoint,"spline_age2:cov21TRUE"] * splinepred[2] +
        fitted_model$cum[timepoint,"spline_age3:cov21TRUE"] * splinepred[3] +
        fitted_model$cum[timepoint,"spline_age4:cov21TRUE"] * splinepred[4]

      c20_cumhaz_age[i] <- cplot20_cumhaz_age
      c21_cumhaz_age[i] <- cplot21_cumhaz_age
```

````
    # covid*wlz-verblijf hazard
    eeplot20_cumhaz_age <- fitted_model$cum[timepoint,"spline_age1:in_wlz_verblijf:cov20TRUE"] *
splinepred[1] +
     fitted_model$cum[timepoint,"spline_age2:in_wlz_verblijf:cov20TRUE"] * splinepred[2] +
     fitted_model$cum[timepoint,"spline_age3:in_wlz_verblijf:cov20TRUE"] * splinepred[3] +
     fitted_model$cum[timepoint,"spline_age4:in_wlz_verblijf:cov20TRUE"] * splinepred[4]

    eeplot21_cumhaz_age <- fitted_model$cum[timepoint,"spline_age1:in_wlz_verblijf:cov21TRUE"] *
splinepred[1] +
     fitted_model$cum[timepoint,"spline_age2:in_wlz_verblijf:cov21TRUE"] * splinepred[2] +
     fitted_model$cum[timepoint,"spline_age3:in_wlz_verblijf:cov21TRUE"] * splinepred[3] +
     fitted_model$cum[timepoint,"spline_age4:in_wlz_verblijf:cov21TRUE"] * splinepred[4]

    ee20_cumhaz_age[i] <- eeplot20_cumhaz_age
    ee21_cumhaz_age[i] <- eeplot21_cumhaz_age

  }

 df_plot <- data.frame(cbind(age = agevec, b_cumhaz_age, wlz_cumhaz_age, c20_cumhaz_age,
c21_cumhaz_age, ee20_cumhaz_age, ee21_cumhaz_age))
 return(df_plot)
}
```

```{r}
my_age <- 70:100

## TIMEPOINT 1 ##
# time-variable for 2020 and 2021

my_time <- 100
my_timepoint <- which.min(abs(fitadd_agespline_full_analysis_from70cM$cum[,1] - my_time))

df_plotW <- GetAgeEffect(agevec = my_age, timepoint = my_timepoint, fitted_model =
fitadd_agespline_full_analysis_from70cW, spline_age = spline_age)
df_plotW$sex <- "Women"
df_plotM <- GetAgeEffect(agevec = my_age, timepoint = my_timepoint, fitted_model =
fitadd_agespline_full_analysis_from70cM, spline_age = spline_age)
df_plotM$sex <- "Men"

df_plotage_agespline3_t100 <- rbind(df_plotW, df_plotM)

p.agespline.age.effect.t100 <- df_plotage_agespline3_t100 %>%
 ggplot(aes(x = age)) +
 geom_line(aes(y = b_cumhaz_age, col = "blue")) +
 geom_line(aes(y = wlz_cumhaz_age, col = "orange")) +
 geom_line(aes(y = c20_cumhaz_age, col = "purple")) +
 geom_line(aes(y = c21_cumhaz_age, col = "purple4")) +
 geom_line(aes(y = ee20_cumhaz_age, col = "red")) +
 geom_line(aes(y = ee21_cumhaz_age, col = "red4")) +
 facet_grid(~sex) +
 labs(x = "Age", y = "Cumulative hazard", title = "Age effect (splines, knots at 75, 85, 95), time = day 100")
+
 scale_color_manual(name = "Mortality components",
             values = c("orange" = "orange", "blue" = "blue", "purple" = "purple", "purple4" = "purple4",
"red" = "red", "red4" = "red4"),
````

```
                labels = c("blue" = "Background", "orange" = "Excess: care home stay", "purple"= "Excess:
Covid-19 pandemic (2020)","purple4"="Excess: Covid-19 pandemic (2021)", "red"= "Excess: excess
(2020)", "red4"= "Excess: excess (2021)"))

p.agespline.age.effect.t100
ggsave(p.agespline.age.effect.t100, file = "../figures/p.agespline.age.effect.t100.full_analysis_from70c.png",
width=10,height=4,dpi=300)


## TIMEPOINT 2 ##
# time-variable for 2020 and 2021
my_time <- 200
my_timepoint <- which.min(abs(fitadd_agespline_full_analysis_from70cM$cum[,1] - my_time))

df_plotW <- GetAgeEffect(agevec = my_age, timepoint = my_timepoint, fitted_model =
fitadd_agespline_full_analysis_from70cW, spline_age = spline_age)
df_plotW$sex <- "Women"
df_plotM <- GetAgeEffect(agevec = my_age, timepoint = my_timepoint, fitted_model =
fitadd_agespline_full_analysis_from70cM, spline_age = spline_age)
df_plotM$sex <- "Men"

df_plotage_agespline3_t200 <- rbind(df_plotW, df_plotM)

p.agespline.age.effect.t200 <- df_plotage_agespline3_t200 %>%
 ggplot(aes(x = age)) +
 geom_line(aes(y = b_cumhaz_age, col = "blue")) +
 geom_line(aes(y = wlz_cumhaz_age, col = "orange")) +
 geom_line(aes(y = c20_cumhaz_age, col = "purple")) +
 geom_line(aes(y = c21_cumhaz_age, col = "purple4")) +
 geom_line(aes(y = ee20_cumhaz_age, col = "red")) +
 geom_line(aes(y = ee21_cumhaz_age, col = "red4")) +
 facet_grid(~sex) +
 labs(x = "Age", y = "Cumulative hazard", title = "Age effect (splines, knots at 75, 85, 95), time = day 200")
+
 scale_color_manual(name = "Mortality components",
                values = c("orange" = "orange", "blue" = "blue", "purple" = "purple", "purple4" = "purple4",
"red" = "red", "red4" = "red4"),
                labels = c("blue" = "Background", "orange" = "Excess: care home stay", "purple"= "Excess:
Covid-19 pandemic (2020)","purple4"="Excess: Covid-19 pandemic (2021)", "red"= "Excess: excess
(2020)", "red4"= "Excess: excess (2021)"))

p.agespline.age.effect.t200
ggsave(p.agespline.age.effect.t200, file = "../figures/p.agespline.age.effect.t200.full_analysis_from70c.png",
width=10,height=4,dpi=300)

## TIMEPOINT 3 ##
# time-variable for 2020 and 2021
my_time <- 300
my_timepoint <- which.min(abs(fitadd_agespline_full_analysis_from70cM$cum[,1] - my_time))

df_plotW <- GetAgeEffect(sexvar = 0, agevec = my_age, timepoint = my_timepoint, fitted_model =
fitadd_agespline_full_analysis_from70cW, spline_age = spline_age)
df_plotW$sex <- "Women"
df_plotM <- GetAgeEffect(sexvar = 1, agevec = my_age, timepoint = my_timepoint, fitted_model =
fitadd_agespline_full_analysis_from70cM, spline_age = spline_age)
df_plotM$sex <- "Men"

df_plotage_agespline3_t300 <- rbind(df_plotW, df_plotM)
```

p.agespline.age.effect.t300 <- df_plotage_agespline3_t300 %>%
  ggplot(aes(x = age)) +
  geom_line(aes(y = b_cumhaz_age, col = "blue")) +
  geom_line(aes(y = wlz_cumhaz_age, col = "orange")) +
  geom_line(aes(y = c20_cumhaz_age, col = "purple")) +
  geom_line(aes(y = c21_cumhaz_age, col = "purple4")) +
  geom_line(aes(y = ee20_cumhaz_age, col = "red")) +
  geom_line(aes(y = ee21_cumhaz_age, col = "red4")) +
  facet_grid(~sex) +
  labs(x = "Age", y = "Cumulative hazard", title = "Age effect (splines, knots at 75, 85, 95), time = day 300")
+
  scale_color_manual(name = "Mortality components",
                values = c("orange" = "orange", "blue" = "blue", "purple" = "purple", "purple4" = "purple4",
"red" = "red", "red4" = "red4"),
                labels = c("blue" = "Background", "orange" = "Excess: care home stay", "purple"= "Excess:
Covid-19 pandemic (2020)","purple4"="Excess: Covid-19 pandemic (2021)", "red"= "Excess: excess
(2020)", "red4"= "Excess: excess (2021)"))

p.agespline.age.effect.t300
ggsave(p.agespline.age.effect.t300, file = "../figures/p.agespline.age.effect.t300.full_analysis_from70c.png",
width=10,height=4,dpi=300)
```

# Model 5: ignoring care home variable, age effect splines (knots 80, 90)

Analysis to illustrate how the background hazard would change if it would be based on the entire population, instead of non-care home residents only.

```{r}
my_knots <- c(80,90)
my_boundary_knots <- c(70, 100) # necessary

spline_age <- ns(data_use_subset$age_floor, knots = my_knots, Boundary.knots = my_boundary_knots)
```

M: Fit model
```{r, eval = F}
spline_age <- ns(data_use_subset$age_floor, knots = my_knots, Boundary.knots = my_boundary_knots)
spline_age <- spline_age[data_use_subset$sex == "male",] # temporary, to get correct covariate names in
model
gc()

fitadd_agespline_full_analysis_from70M_nowlz <- aalen(Surv(entry, srv, srv_s) ~
                        spline_age +
               cov20 + cov20:spline_age +
               cov21 + cov21:spline_age,
                 data = data_use_subset[data_use_subset$sex == "male",], robust = 0)


save(fitadd_agespline_full_analysis_from70M_nowlz, file =
"../objects/fitadd_agespline_full_analysis_from70M_nowlz.RData")
spline_age <- ns(data_use_subset$age_floor, knots = my_knots, Boundary.knots = my_boundary_knots)
```

W: Fit model
```{r, eval = F}
spline_age <- ns(data_use_subset$age_floor, knots = my_knots, Boundary.knots = my_boundary_knots)
spline_age <- spline_age[data_use_subset$sex == "female",] # temporary, to get correct covariate names in
model

```
gc()

fitadd_agespline_full_analysis_from70W_nowlz <- aalen(Surv(entry, srv, srv_s) ~
                spline_age +
        cov20 + cov20:spline_age +
        cov21 + cov21:spline_age,
          data = data_use_subset[data_use_subset$sex == "female",], robust = 0)


save(fitadd_agespline_full_analysis_from70W_nowlz, file = "../objects/fitadd_agespline_full_analysis_from70W_nowlz.RData")
spline_age <- ns(data_use_subset$age_floor, knots = my_knots, Boundary.knots = my_boundary_knots)
```

Plot results

```{r}
load("H:/DECIM_Marije/excess_excess/additive_hazards/objects/fitadd_agespline_full_analysis_from70M_nowlz.RData")
load("H:/DECIM_Marije/excess_excess/additive_hazards/objects/fitadd_agespline_full_analysis_from70W_nowlz.RData")
```

```{r}
GetHazards <- function(agevar, fitted_model, splinepred){

  # time-variable for 2020 and 2021
  tplot20 <- fitted_model$cum[,1]
  tplot21 <- tplot20 + max(tplot20) - 1 # -1 because Jan 1, 2021 is the 367th day of my model (not the 368th)
  tplot <- c(tplot20[-1], tplot21[-1]) # first element not counted because that is 'day 0' when I let everyone enter but no one die
  tplot <- tplot[-length(tplot)] # remove last element because 2021 only has 365 days

  #   background hazard: intercept + sex + age + age:sex
  bplot20 <- fitted_model$cum[,"(Intercept)"] +
   fitted_model$cum[,"spline_age1"] * splinepred[1] +
   fitted_model$cum[,"spline_age2"] * splinepred[2] +
   fitted_model$cum[,"spline_age3"] * splinepred[3]

  bplot21 <- bplot20 + tail(bplot20, 1)
  bplot <- c(bplot20[-1], bplot21[-1])
  bplot <- bplot[-length(bplot)]

  #   covid-19 hazard: covid20-main, covid20:age_cat, covid20:sex, covid20:age_cat:sex (same for covid21)
  cplot20 <- fitted_model$cum[,"cov20TRUE"] +
   fitted_model$cum[,"spline_age1:cov20TRUE"] * splinepred[1] +
   fitted_model$cum[,"spline_age2:cov20TRUE"] * splinepred[2] +
   fitted_model$cum[,"spline_age3:cov20TRUE"] * splinepred[3]

  c21 <- fitted_model$cum[,"cov21TRUE"] +
   fitted_model$cum[,"spline_age1:cov21TRUE"] * splinepred[1] +
   fitted_model$cum[,"spline_age2:cov21TRUE"] * splinepred[2] +
   fitted_model$cum[,"spline_age3:cov21TRUE"] * splinepred[3]

  cplot21 <- c21 + tail(cplot20, n = 1)
  cplot <- c(cplot20[-1], cplot21[-1])
  cplot <- cplot[-length(cplot)]
```

````
   df_plot <- data.frame(tplot = tplot,
                   bplot = bplot,
                   cplot = cplot,
                   age = agevar)

   return(df_plot)
}
```

```{r}
my_agevars = c(75, 85, 95)

## men ##

df_plot_listM <- list()

for (i in 1:length(my_agevars)){

  my_splinepred <- predict(spline_age, my_agevars[i])[1,]
  df_plot <- GetHazards(agevar = my_agevars[i], fitted_model =
fitadd_agespline_full_analysis_from70M_nowlz, splinepred = my_splinepred)
  df_plot_listM[[i]] <- df_plot
}

df_plot_list_coarsenedM <- lapply(df_plot_listM, Coarsen, n_timepoints = 2000)
df_plot_nowlzM <- list_rbind(df_plot_list_coarsenedM)

df_plot_nowlzM$sex <- "Men"

## women ##

df_plot_listW <- list()

for (i in 1:length(my_agevars)){

  my_splinepred <- predict(spline_age, my_agevars[i])[1,]
  df_plot <- GetHazards(agevar = my_agevars[i], fitted_model =
fitadd_agespline_full_analysis_from70W_nowlz, splinepred = my_splinepred)
  df_plot_listW[[i]] <- df_plot
}

df_plot_list_coarsenedW <- lapply(df_plot_listW, Coarsen, n_timepoints = 2000)
df_plot_nowlzW <- list_rbind(df_plot_list_coarsenedW)

df_plot_nowlzW$sex <- "Women"

df_plot_nowlz <- rbind(df_plot_nowlzM, df_plot_nowlzW)

p.bhaz.grid.agespline.nowlz.comparison <- df_plot_nowlz %>%
  ggplot(aes(x = tplot)) +
  geom_line(aes(y = bplot, col = "grey")) +
  geom_line(aes(y = bplot, col = "blue"), data = df_plothaz_agespline2) +
  labs(x = "Days (2020 + 2021)", y = "Cumulative hazard", title = "Age with splines (knots at 75, 90)") +
  scale_color_manual(name = "Mortality components", values = c("blue" = "blue", "grey" = "grey"), labels =
c("Background [0,0]-group", "Background whole population (lifetable)")) +
  facet_grid(sex~age) +
  coord_cartesian(ylim = c(0, 0.75))
p.bhaz.grid.agespline.nowlz.comparison
````

ggsave(p.bhaz.grid.agespline.nowlz.comparison, file =
"../figures/p.allhaz.grid.agespline.nowlz.comparison.png", width=10,height=4,dpi=300)
```

# Saving for export CBS

```{r}
write.xlsx(df_plothaz_agecat, file = "../data_export/final/df_plothaz_agecat.xlsx", row.names = F)
save(df_plothaz_agecat, file = "../objects/final/df_plothaz_agecat.RData")
save(df_plotage_agecat_t100, file = "../objects/final/df_plotage_agecat_t100.RData")
save(df_plotage_agecat_t200, file = "../objects/final/df_plotage_agecat_t200.RData")
save(df_plotage_agecat_t300, file = "../objects/final/df_plotage_agecat_t300.RData")

df_plothaz_agespline2$bplot_nowlz <- df_plot_nowlz$bplot
df_plothaz_agespline2$cplot_nowlz <- df_plot_nowlz$cplot
write.xlsx(df_plothaz_agespline2, file = "../data_export/final/df_plothaz_agespline2.xlsx", row.names = F)
save(df_plothaz_agespline2, file = "../objects/final/df_plothaz_agespline2.RData")
save(df_plotage_agespline2_t100, file = "../objects/final/df_plotage_agespline2_t100.RData")
save(df_plotage_agespline2_t200, file = "../objects/final/df_plotage_agespline2_t200.RData")
save(df_plotage_agespline2_t300, file = "../objects/final/df_plotage_agespline2_t300.RData")

write.xlsx(df_plothaz_agespline1, file = "../data_export/final/df_plothaz_agespline1.xlsx", row.names = F)
save(df_plothaz_agespline1, file = "../objects/final/df_plothaz_agespline1.RData")
save(df_plotage_agespline1_t100, file = "../objects/final/df_plotage_agespline1_t100.RData")
save(df_plotage_agespline1_t200, file = "../objects/final/df_plotage_agespline1_t200.RData")
save(df_plotage_agespline1_t300, file = "../objects/final/df_plotage_agespline1_t300.RData")

write.xlsx(df_plothaz_agespline3, file = "../data_export/final/df_plothaz_agespline3.xlsx", row.names = F)
save(df_plothaz_agespline3, file = "../objects/final/df_plothaz_agespline3.RData")
save(df_plotage_agespline3_t100, file = "../objects/final/df_plotage_agespline3_t100.RData")
save(df_plotage_agespline3_t200, file = "../objects/final/df_plotage_agespline3_t200.RData")
save(df_plotage_agespline3_t300, file = "../objects/final/df_plotage_agespline3_t300.RData")

```
```
